# MLL1 is essential for retinal neurogenesis and horizontal inner neuron integrity

**DOI:** 10.1038/s41598-018-30355-3

**Published:** 2018-08-09

**Authors:** Diana S. Brightman, Rachel L. Grant, Philip A. Ruzycki, Ray Suzuki, Anne K. Hennig, Shiming Chen

**Affiliations:** 1 Department of Ophthalmology and Visual Sciences, Washington University, Saint Louis, Missouri USA; 2 Department of Developmental Biology, Washington University, Saint Louis, Missouri USA; 3 Molecular Cell Biology graduate program, Division of Biology & Biomedical Sciences, Washington University, Saint Louis, Missouri USA; 4 Molecular Genetics and Genomics graduate program, Division of Biology & Biomedical Sciences, Washington University, Saint Louis, Missouri USA; 5 College of Arts and Sciences, Washington University, Saint Louis, Missouri USA; 6 Present Address: Cincinnati Children’s Hospital Medical Center, 3333 Burnet Avenue, ML 4006, Cincinnati, OH 45229 USA

## Abstract

Development of retinal structure and function is controlled by cell type-specific transcription factors and widely expressed co-regulators. The latter includes the mixed-lineage leukemia (MLL) family of histone methyltransferases that catalyze histone H3 lysine 4 di- and tri-methylation associated with gene activation. One such member, MLL1, is widely expressed in the central nervous system including the retina. However, its role in retinal development is unknown. To address this question, we knocked out *Mll1* in mouse retinal progenitors, and discovered that MLL1 plays multiple roles in retinal development by regulating progenitor cell proliferation, cell type composition and neuron-glia balance, maintenance of horizontal neurons, and formation of functional synapses between neuronal layers required for visual signal transmission and processing. Altogether, our results suggest that MLL1 is indispensable for retinal neurogenesis and function development, providing a new paradigm for cell type-specific roles of known histone modifying enzymes during CNS tissue development.

## Introduction

The vertebrate retina is a central nervous system structure specialized for vision. Six major classes of neurons and one type of glia (Muller) are organized into three cell layers^1^. The outer nuclear layer (ONL) contains rod and cone photoreceptors, which convert light into a neuronal signal. The inner nuclear layer (INL) contains cell bodies of Muller glia and bipolar, horizontal and amacrine interneurons, which mediate transmission and initial processing of the visual signal. Ganglion cells in the ganglion cell layer (GCL) transmit the processed visual information to the brain via the optic nerve. These three cellular layers are connected by two synaptic layers, the outer and inner plexiform layers (OPL and IPL).

Although retina development occurs at different rates in different species, genesis of individual cell types follows a highly conserved order^2^. As the retina develops, multipotent progenitor cells become more lineage-restricted^3^. In mice, retinal neurogenesis occurs between embryonic day E11 and postnatal day P10, prior to eye opening at P14. The early retinal progenitor cells that exit the cell cycle during embryonic (prenatal) development give rise to ganglion, horizontal, cone and some amacrine cells, while later progenitor cells that stop dividing during postnatal development give rise to rod, bipolar, late-born amacrine cells and Muller glia^4^.

Retinogenesis is governed by a genetic program that integrates extrinsic signals to precisely control spatial and temporal patterning of the retina^4,5^. This genetic program is built on a network of lineage-specific transcription factors (TF), many of which are multifunctional and act at specific developmental time points. Several homeodomain TFs, such as RAX^6^, PAX6^7^ and CHX10^8,9^, are expressed by progenitor cells to maintain multipotency. Other TFs specify particular cell types: OTX2^10^, CRX^11,12^ and NRL^13^ specify rod cell fate, while Onecut1 (OC1)^14^, PROX1^15^ and LIM1^16^ are involved in horizontal cell development.

Lineage-specific transcription factors also interact with widely-expressed co-regulators to modulate chromatin accessibility for target gene regulation. These co-regulators include enzymes that catalyze post-translational modifications of histone tails (“histone marks”). Active genes usually carry the positive histone marks H3K4me3 and H3K27Ac, while silent genes are often marked by H3K9me3 and H3K27me3^17,18^.

The histone lysine methyltransferases (KMTs) that “write” the positive marks H3K4me1–3 include the members of mixed-lineage leukemia (MLL) family: MLL1 (KMT2A), MLL2 (KMT2B), MLL3 (KMT2C) and MLL4 (KMT2D). All contain the conserved catalytic SET domain and form large multi-protein complexes to remodel the epigenome^19–21^. Initially discovered through their association with cancer^22,23^, MLLs are essential for organ/tissue genesis. Germline knockout of individual MLLs causes embryonic lethality^24–28^. Targeted inactivation of MLL family members in various cell types has revealed diverse roles in developing^27–33^ and adult animals^21^.

Among the four members, MLL1 (MLL in human) is the most extensively studied. In mice, MLL1 is required for hematopoiesis^29^ and neurogenesis in the postnatal brain, where it regulates neural progenitor proliferation and cell fate specification^33^. MLL1 also plays a role in synaptic plasticity and memory^31,34,35^. In zebrafish, MLL1 is necessary for neural development and progenitor proliferation^36^, suggesting a conserved role in CNS development. However, the role of MLL1 in the development of mammalian sensory neurons, particularly in the visual system, is completely unknown. Using conditional knockout strategies, we have found that MLL1 is essential for retinal structure and function development, particularly in progenitor cell proliferation, cell type composition and neuron-glia balance, horizontal cell differentiation and maintenance, and functional synapse formation. Our study uncovers specific roles of MLL1 in sensory neuronal tissue development, supporting the concept that a general histone modifying enzyme can contribute to cell-type-specific transcriptional regulation.

## Results

### Mll1 is expressed in all neuronal layers of the mouse retina

**Figure 1:**
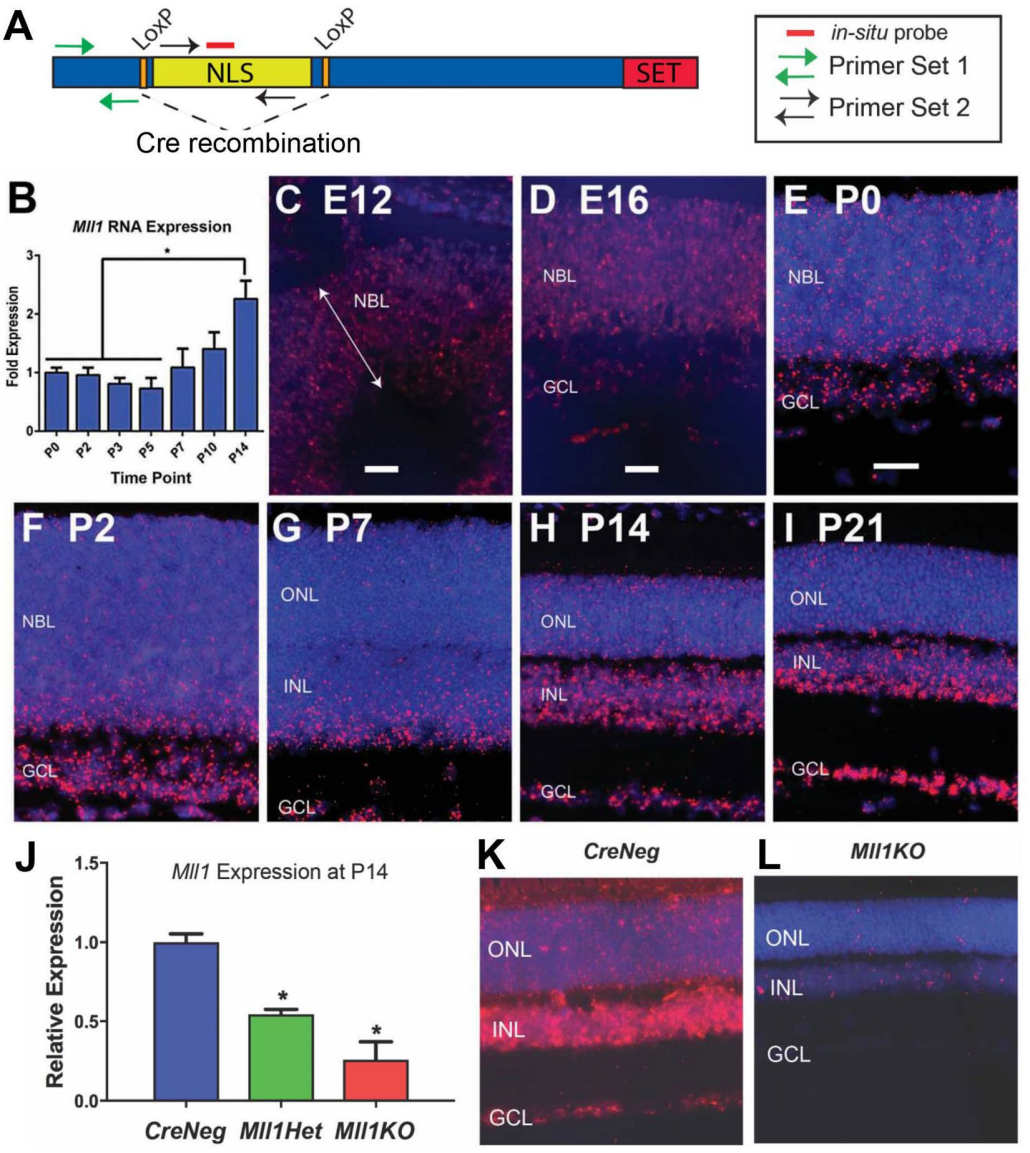
*Mll1* expression in mouse retina and its conditional knockout. (**A**) Diagram of mouse *Mll1* coding sequence showing the positions of the catalytic SET domain and the *LoxP* sites flanking the nuclear localization signals encoded in exons 3-4^29^. The binding sites for the *in situ* probe (red solid line) and two pairs of RT-PCR primers (arrows; sequences given in Supplementary Table S1) are indicated. (**B**) Quantitative RT-PCR analyses of *Mll1* mRNA from the indicated postnatal ages using Primer Set 1, presented as fold expression relative to P0 samples, with error bars representing standard error of mean (SEM, n ≥ 3). *p < 0.05 based on one-way ANOVA with Tukey’s multiple comparisons. (**C–I**) *in situ* hybridization of *Mll1* (red) with DAPI nuclear stain (blue) in retinal cross-sections of wild type (*C57BL/6 J*) mice at the indicated developmental time points. Note that the signal intensity increases in two inner retinal layers at P14 compared to earlier ages. NBL-neuroblast layer; GCL-ganglion cell layer; ONL-outer nuclear layer; INL-inner nuclear layer. Scale bar = 25um for Panels C, D, and E-I. (**J**) Quantitative RT-PCR analyses of *Mll1* mRNA expression using Primer Set 2. The results are presented as fold expression relative to *CreNeg* littermate samples, with error bars representing SEM (n ≥ 3). *p < 0.05 compared to *CreNeg* by one-way ANOVA with Tukey’s multiple comparisons. (**K**,**L**) *In situ* hybridization with *Mll1* probe on retinal cross-sections of 1-month old *CreNeg* and *Mll1KO* mice.

To determine spatial and temporal patterns of *Mll1* expression in the mouse retina, we analyzed *Mll1* transcript levels and distribution during development. Quantitative real-time PCR (qRT-PCR) assays with *Mll1*-specific “Primer Set 1” (Fig. 1A, Supplementary Table S1) showed that *Mll1* expression was constant from Postnatal day 0 (P0) to P5, but significantly increased in the second postnatal week (Fig. 1B), the crucial period for terminal differentiation of retinal neurons. *In situ* hybridization (ISH) with a *Mll1* probe (Fig. 1A) showed *Mll1* transcripts detectable as early as Embryonic Day 12 (E12), distributed uniformly across the entire neuroblast layer (NBL, Fig. 1C) where neurogenesis occurs. At E16, *Mll1* was expressed in both the NBL and ganglion cell layer (GCL, Fig. 1D). *Mll1* expression was continuously detected in all layers of postnatal developing and mature mouse retinas from P0 to P21 (Fig. 1E–I). Consistent with qRT-PCR results (Fig. 1B), the ISH signal intensity was much higher at P14 compared to P7 and earlier, particularly in the inner nuclear layer (INL) and GCL (Fig. 1H,G). At P14, *Mll1* expression was also seen in the outer nuclear layer (ONL) where differentiated photoreceptor nuclei reside, although the signal was sparse and much weaker than INL and GCL (Fig. 1H). This pattern of *Mll1* expression remained at P21 (Fig. 1I) and throughout adulthood. Overall, *Mll1* is widely expressed in neural progenitors, developing and differentiated neurons, particularly in the inner retina.

### Generation of *Mll1* conditional knockout in developing retinas

To investigate the role of MLL1 in retinal development we conditionally knocked out *Mll1* in developing mouse retinas using Cre*-LoxP* mediated recombination. We crossed mice carrying *Mll1 flox* alleles^29^ (Fig. 1A) with a *Chx10-Cre* line that expresses Cre recombinase in progenitor cells throughout retinal development^37^. To confirm successful recombination of the *Mll1* floxed region, we performed qRT-PCR for unrecombined *Mll1* RNA using “Primer Set 2” located in the CRE-excised region (Fig. 1A). At P14, the retinas of heterozygous (*Mll1Het*) and homozygous (*Mll1KO*) knockout mice expressed 55% and 26% of normal amounts of *Mll1* mRNA, respectively (Fig. 1J). To reveal the extent of *Mll1* knockout (KO) at the cellular level, we performed *in situ* hybridization (Fig. 1K,L) with a probe specific for unrecombined *Mll1* RNA (Fig. 1A). Compared to *CreNeg* littermates (Fig. 1K), the majority of retinal cells in *Mll1KO* retinas no longer bound probe, although a few cells remained positive for *Mll1* transcript (Fig. 1L). We thus refer to the homozygous *Mll1*^*f/f*^*; Chx10-Cre* mice as *Mll1KO* (or *KO*).

### *Mll1* deficiency causes deficits in retinal function

**Figure 2:**
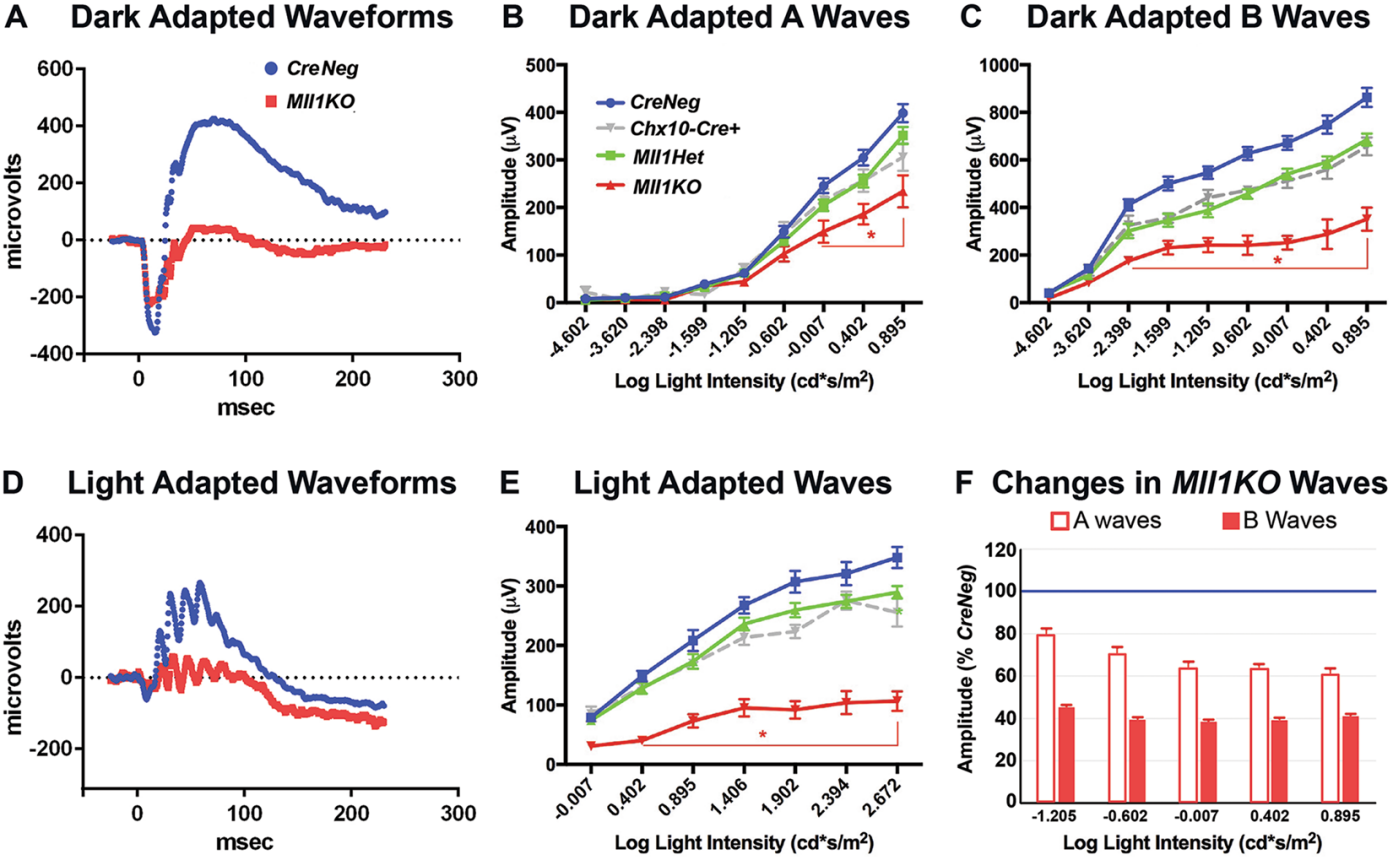
Electroretinogram (ERG) analyses of *Mll1KO* mice and littermate controls. Whole animal dark-adapted (**A–C**) and light-adapted (**D**,**E**) flash ERGs were performed on 1MO mice. (**A**,**D**) Example waveforms in response to the brightest light flash intensity tested. (**B**,**C**,**E**) Mean dark-adapted A and B and light-adapted B wave amplitudes were graphed vs. stimulus light intensity. Error bars represent SEM; *p < 0.05 relative to *CreNeg* controls by two-way repeated measures ANOVA and Tukey’s multiple comparisons (n ≥ 4). (**F**) Dark-adapted *Mll1KO* A and B wave amplitudes for the five brightest flash intensities tested were normalized to the respective mean *CreNeg* amplitudes and graphed.

To determine the consequence of *Mll1* deficiency on overall retinal function, we performed electroretinography (ERG) at 1 month of age (1MO). Both dark-adapted and light-adapted response waveforms from *Mll1KO* mice showed A waves, B waves and oscillatory potentials, with latencies comparable to *CreNeg* littermate controls (Fig. 2A,D). However, *Mll1KO* amplitudes were markedly decreased (Fig. 2A–E) across most of the range of light intensities tested under both conditions. Notably, the dark-adapted amplitude reductions were disproportionally greater for B than A waves (Fig. 2F), suggesting that *Mll1KO* retinas not only have rod/cone dysfunction, but also deficits in visual signal transmission from photoreceptors to inner neurons. These defects did not worsen with age up to 6 months (data not shown) arguing against progressive degeneration in this model. Finally, both heterozygous littermates (*Mll1Het*) and *Cre-*expressing mice lacking floxed *Mll1* alleles showed only non-significant minor reductions in their A- and B-wave amplitudes relative to *CreNeg* controls (Fig. 2B,C,E), indicating that *Mll1* deficiency, not *Cre* expression itself, is responsible for *Mll1KO* functional deficits.

### *Mll1* deficiency produces thinner retinas, particularly affecting the inner layers

**Figure 3:**
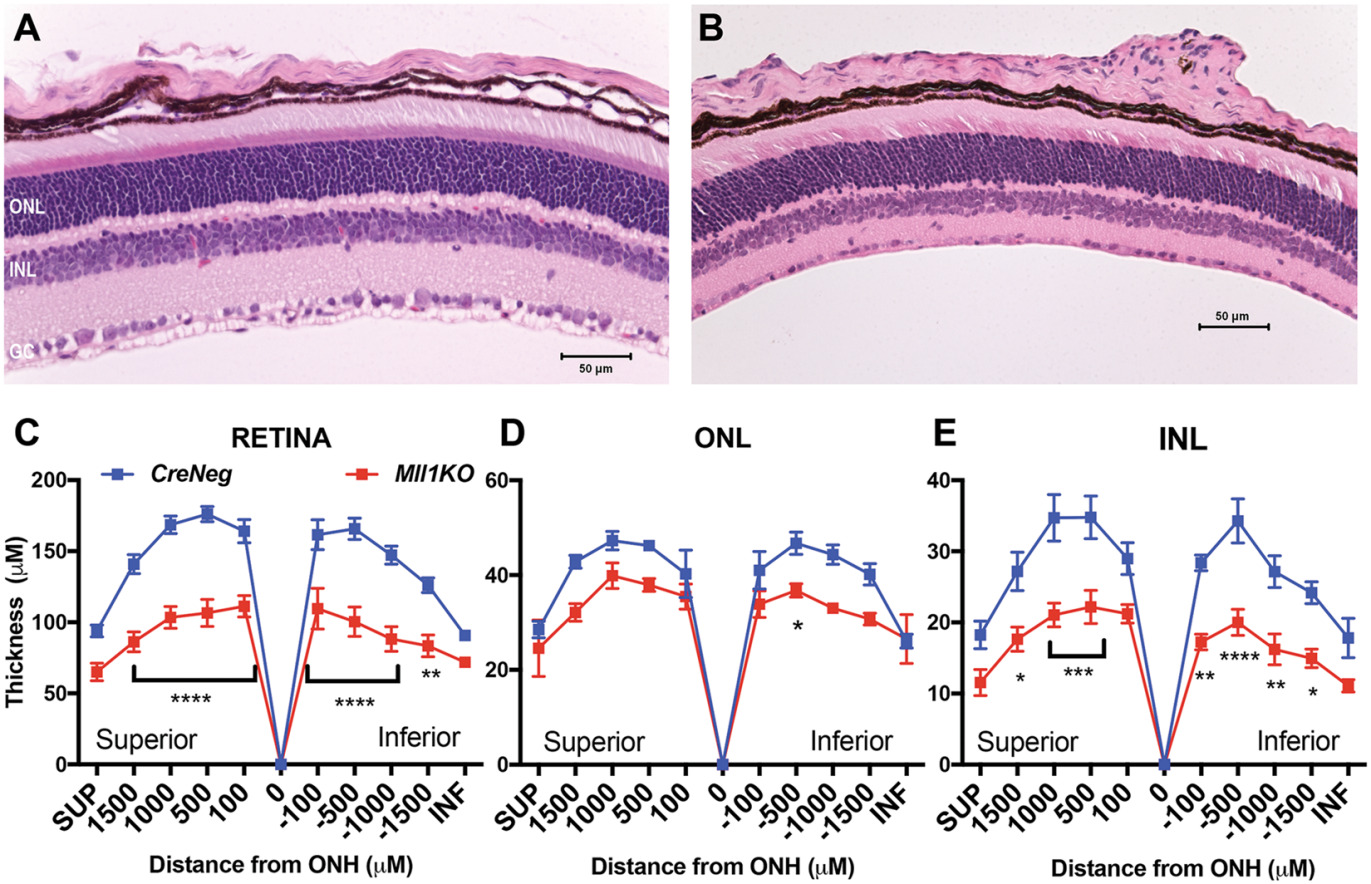
H&E staining of *CreNeg* (**A**) and *Mll1KO* (**B**) retinal cross-sections at P24. (**C–E**) Morphometric quantification of retinal layer thickness at the indicated positions from the optic nerve head (ONH). SUP and INF indicate measurements taken 100um from the superior or inferior edge of the retina. *p < 0.05; **p < 0.01; ***p < 0.001; ****p < 0.0001, based on two-way ANOVA with repeated measures and Sidak’s multiple comparisons test (n = 4). Histologic results at other ages are shown in Supplementary Fig. S1. Cell death in developing retinas assessed by immunostaining for Cleaved Caspase 3 is shown in Supplementary Fig. S2.

To determine if *Mll1KO* visual functional deficits stem from abnormal retinal architecture, we examined hematoxylin-and-eosin (H&E)–stained P24 retinal cross-sections (Fig. 3). *Mll1KO* young adult retinas looked grossly normal with well-separated neuronal layers, but appeared thinner than *CreNeg* controls (Fig. 3B,A). We measured the thickness of the whole retina and of individual nuclear layers at various distances from the optic nerve head (Fig. 3C–E). The overall retinal thickness was significantly reduced on both the superior and inferior side compared to *CreNeg* littermates (Fig. 3C). Although both ONL (Fig. 3D) and INL (Fig. 3E) were thinner in *Mll1KO* retinas, only the INL changes reached statistical significance, suggesting that thickness reduction of *Mll1KO* retinas is largely attributable to decreases in INL cells and their processes/synapses.

We also examined H&E-stained retinal sections at various postnatal ages (Supplementary Fig. S1). At P0, *Mll1KO* retinas were already slightly thinner than *CreNeg* controls (Supplementary Fig. S1A-D). At P7, *Mll1KO* retinas showed normal ONL and INL separation (Supplementary Fig. S1F vs S1E), but were significantly thinner than *CreNeg* controls, particularly the INL (Supplementary Fig. S1G-I). *Mll1KO* retina and INL thickness reductions persisted at P14 (Supplementary Fig. S1J-N) and into young adulthood (Fig. 3). Morphometric analyses performed at 7 months showed a similar pattern (Supplementary Fig. S1O-S), consistent with the relatively constant ERG amplitude reductions. Together, these results suggest that the functional and morphological abnormalities that develop in *Mll1KO* retinas during neurogenesis and terminal differentiation do not trigger retinal degeneration.

To investigate the cause of retinal thinning in *Mll1KO* mice, we immunostained for activated Caspase-3 at five different developmental ages, E16, P0, P3, P7 and P14. The number of apoptotic cells was comparable or decreased compared to controls at all ages (Supplementary Fig. S2). Thus, the thinning of *Mll1KO* retinas is unlikely due to increased cell death.

### *Mll1* deficiency reduces progenitor cell proliferation and cell cycle progression

**Figure 4:**
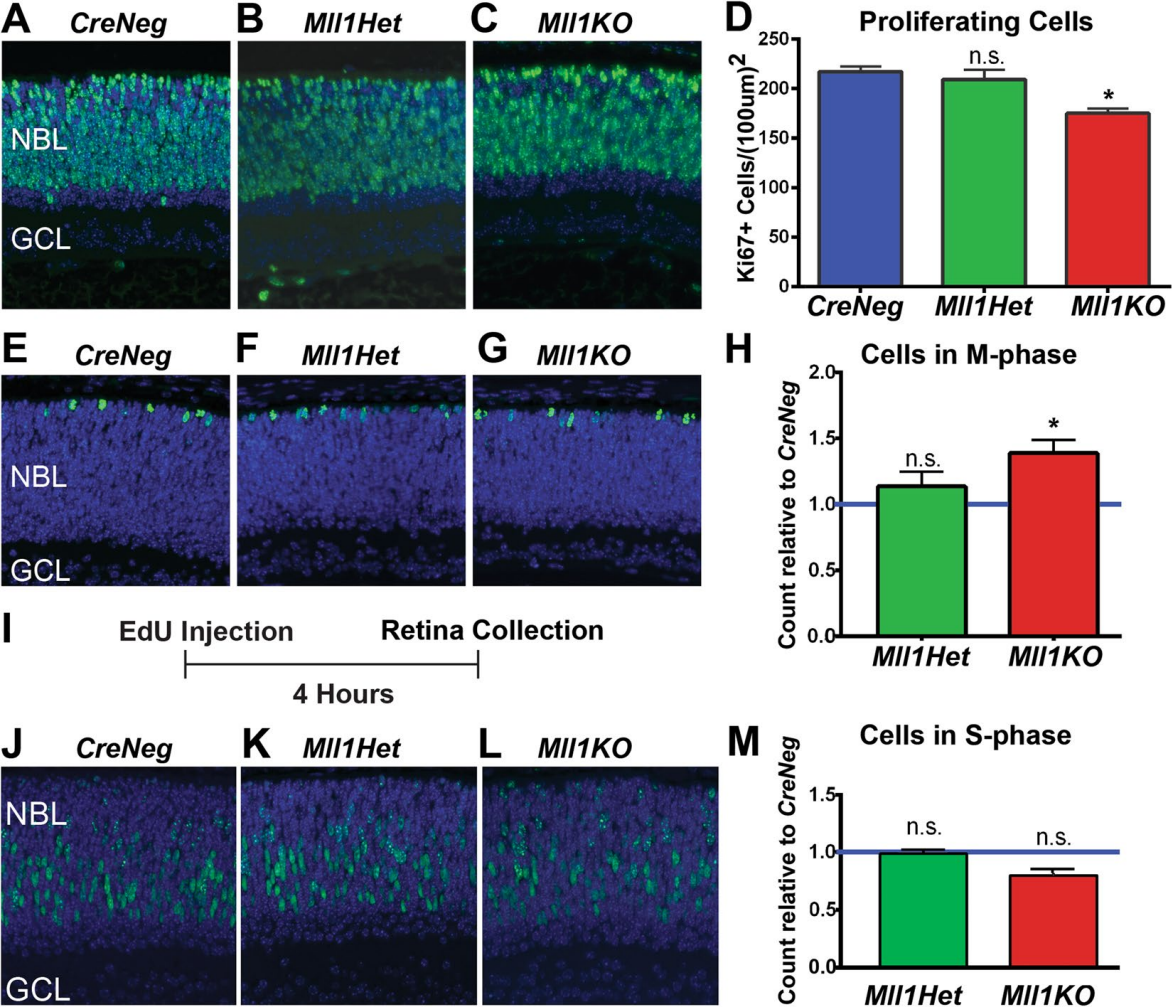
Loss of *Mll1* in retinal progenitor cells results in proliferation and cell cycle defects. (**A-C**) Ki67 immunostaining (green) for proliferative cells with DAPI nuclear stain (blue) of retinal cross-sections in *CreNeg* (**A**), *Mll1Het* (**B**) and *Mll1KO* (**C**) mice at P0. (**D**) Quantification of Ki67 + cells in three 100 μm^2^ regions per cross-section, three cross-sections per sample and three samples per genotype. Results are displayed as mean + SEM (n = 3). (**E–G**) Phospho-histone H3 (PH3) immunostaining (green) with DAPI (blue) of the same samples for cells in mitotic (M-) phase of the cell cycle. (**H**) Quantification of the number of Ki67+ cells positive for PH3 per cross-section, normalized to the number in *CreNeg* sections. The results are displayed as mean + SEM. (**I**) To determine S-phase cells in P0 retinas, EdU was injected IP into P0 mouse pups, and retinas harvested 4 hours later. (**J–L**) EdU labeled cells (green) with DAPI (blue) in retinal cross-sections of harvested retinas. (**M**) Quantification of the number of Ki67+ cells reactive for EdU in three 100 μm^2^ regions per cross-section, relative to the number in *CreNeg* sections. The results are displayed as mean + SEM. *p < 0.05, n.s. = not significant by two-way ANOVA with Tukey’s multiple comparisons (n = 3). Antibody information is given in Supplementary Table S2. Reductions in proliferating cells were also detected in *Mll1KO* retinas at P3 and P5 using EdU pulse-labeling (Supplementary Fig. S3A).

To determine if decreased cell proliferation contributed to *Mll1KO* retinal layer thinning, we performed Ki67 immunostaining for proliferating cells in retinal cross sections of *Mll1KO* and *CreNeg* control mice. Figure 4A–C show representative images taken at P0, when altered retina thickness was initially detected in *Mll1KO* retinas. The number of Ki67+ proliferative cells was moderately decreased in *Mll1KO* but not heterozygous (*Mll1Het*) retinas, compared to *CreNeg* littermates (Fig. 4D). Decreased Ki67+ cells were also observed in *Mll1KO* retinas at P3 and P5 (data not shown) and confirmed by 5-Ethynyl-2′-deoxyuridine (EdU) incorporation experiments (Supplementary Fig. S3A). Thus, *Mll1*-deficient progenitor cells prematurely lose their proliferation potential.

To determine which phase of the cell cycle was affected, first we immunostained P0 retinal sections for phospho-histone H3 (PH3) to mark cells in mitosis (M-phase). PH3+ cells were detected at the outer retinal neuroblast layer (NBL) of all genotypes (Fig. 4E–G). When counted and adjusted for the number of Ki67-labeled proliferating cells, cells in M-phase were slightly but significantly increased in *Mll1KO* retinas relative to *CreNeg* controls, while *Mll1Het* M-phase cells showed no significant changes (Fig. 4H). Next, we assessed S-phase cell numbers by EdU pulse labeling (Fig. 4I): After a 4-hour pulse, labeled cells were mostly distributed in the inner part of the NBL, and *Mll1KO* retinas had fewer S-phase cells than *CreNeg* and *Mll1Het* retinas (Fig. 4J–L). Because of differences in Ki67+ cell numbers between genotypes, we counted the number of cells positive for both Ki67 and EdU to determine whether the proportions of proliferating cells in S-phase differed. Both *Mll1KO* and *Mll1Het* proliferating progenitor cell populations had comparable percentages of S-phase cells to *CreNeg* controls (Fig. 4M). This disproportionate M-phase cell increase suggests that MLL1 regulates M-phase progression in retinal progenitors.

MLL1 is known to be required for normal cell cycle progression in cultured cells by regulating cyclins, cyclin-dependent kinases (CDKs) or inhibitors^38^. To test if cyclin-related genes are expressed normally in neonatal retinas of *Mll1KO* mice, we immunostained P0 retinas for cyclin D1 (CCND1)^39,40^ and the CDK inhibitor P27^Kip1^^41,42^, both of which regulate appropriate RPC cycling in developing retinas. The number of CCND1-positive cells was comparable in P0 *Mll1KO* vs. *CreNeg* control retinas (Supplementary Fig. S3B,C), suggesting that *Mll1KO* retinas retain the capability to express *CCND1* during the cell cycle, at least in P0 retinas. However, the number of P27^Kip1^-positive cells was significantly lower in *Mll1KO* than in *CreNeg* controls (Supplementary Fig. S3D,E). Since P27^Kip1^ is expressed both in cycling cells and transiently in all post-mitotic retinal lineages^42^, this reduction likely reflects the decreased number of proliferating cells and their progeny in *Mll1*-deficient retinas (Fig. 4D). These results support the reasoning that early postnatal *Mll1KO* retinas exhibit a defect in RPC proliferation and cell cycle progression that does not directly impair expression of Cyclin D1.

### *Mll1* deficiency alters retinal cell composition and neuron-to-glia ratio

**Figure 5:**
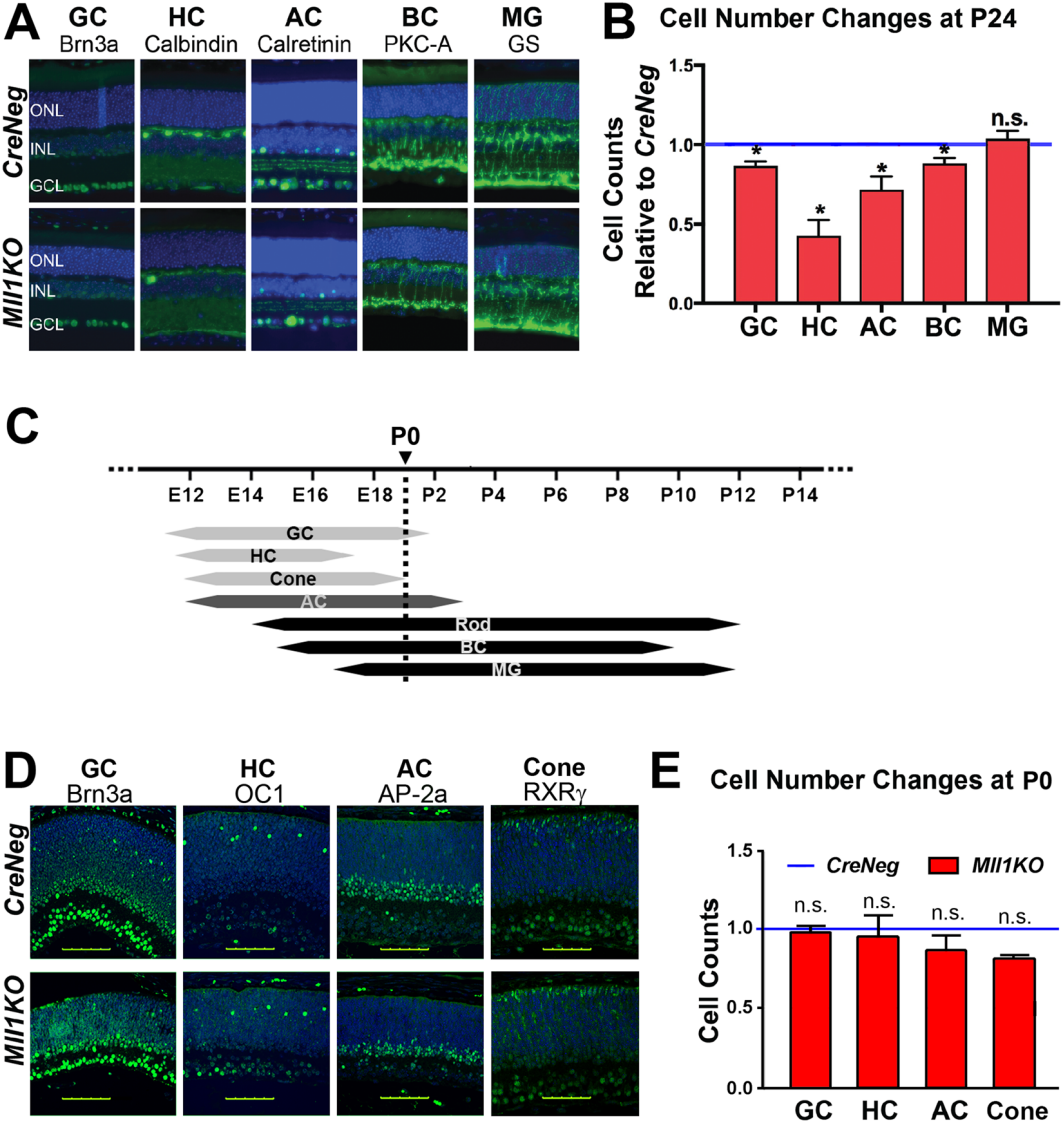
*Mll1* deficiency alters the retinal cell type composition at P24 but not at P0. (**A**) Immunostaining of retinal cross-sections from P24 *CreNeg* and *Mll1KO* mice with antibodies to specific markers for inner retinal cell types (green): Brn3a for Ganglion Cells (GC); Calbindin for Horizontal Cells (HC); Calretinin for starburst Amacrine Cells (AC); Protein Kinase C-alpha (PKC- A) for On-Bipolar Cells (BC); Glutamine Synthetase (GS) for Muller Glia (MG). Antibody information is given in Supplementary Table S2. (**B**) Cell count changes of each inner retinal cell type in *Mll1KO* retinas, presented as mean cell number relative to *CreNeg*, with error bars representing standard error of mean (SEM, n = 4). *p < 0.01, n.s. = not significant by two-way ANOVA. **(C)** Schematic diagram showing when progenitors of each retinal cell type exit the cell cycle. Early-born cell types include GC, HC, and Cone (light gray arrows); late-born cell types include Rod, BC, and MG (black bars). Different subtypes of amacrine cells (ACs) are born at both early and late times (indicated by dark gray bar). (**D**) Immunostaining of P0 retinal cross-sections for markers of early-born cell types: Brn3a for GC, Onecut1 (OC1) for HC, AP-2 alpha (AP-2a) for early-born AC and RXRγ for Cone. (**E**) The number of cells reactive for each marker per retina cross-section was counted. Results are presented as mean cell number relative to *CreNeg* with error bars representing SEM. n.s. = not significant by two-way ANOVA with Tukey’s multiple comparisons (n = 3).

To determine if retinal progenitor cell loss affects cell type composition in mature *Mll1KO* retinas, we quantified early vs. late-born cell types in P24 retina cross-sections after cell differentiation is complete. We focused on cells of the inner retina, since this layer is significantly affected in *Mll1KO* retinas (Fig. 3). Figure 5A shows retinal sections stained with markers for ganglion (GC), horizontal (HC), starburst amacrine (AC) and rod ON-bipolar (BC) neurons and Muller glia (MG). Each neuronal cell type was present but decreased in *Mll1KO* compared to *CreNeg* retinas, with HC and AC populations affected the most (Fig. 5A,B). In contrast, MGs were present in comparable numbers.

To determine the source of these cell composition changes, we first investigated whether retinal progenitor cells (RPCs) produced the expected numbers of committed neuronal precursors. During mouse development, RPCs generate each of the seven major retinal cell types within a defined period (Fig. 5C)^2,3^. We began by staining P0 retinas for specific transcription factors in different early-born cell types, GC (Brn3a), HC (OC1), early-born AC (AP2α) and Cone (RXRγ) (Fig. 5D). Cell counts showed that, at P0, the numbers of each early-born cell type are comparable in *Mll1KO* vs. *CreNeg* control retinas (Fig. 5E), suggesting that early RPC-mediated neurogenesis is largely unaffected in *Mll1*-deficient retinas. Thus, unknown post-neurogenic mechanisms must account for the loss of early-born cells at later ages.

**Figure 6:**
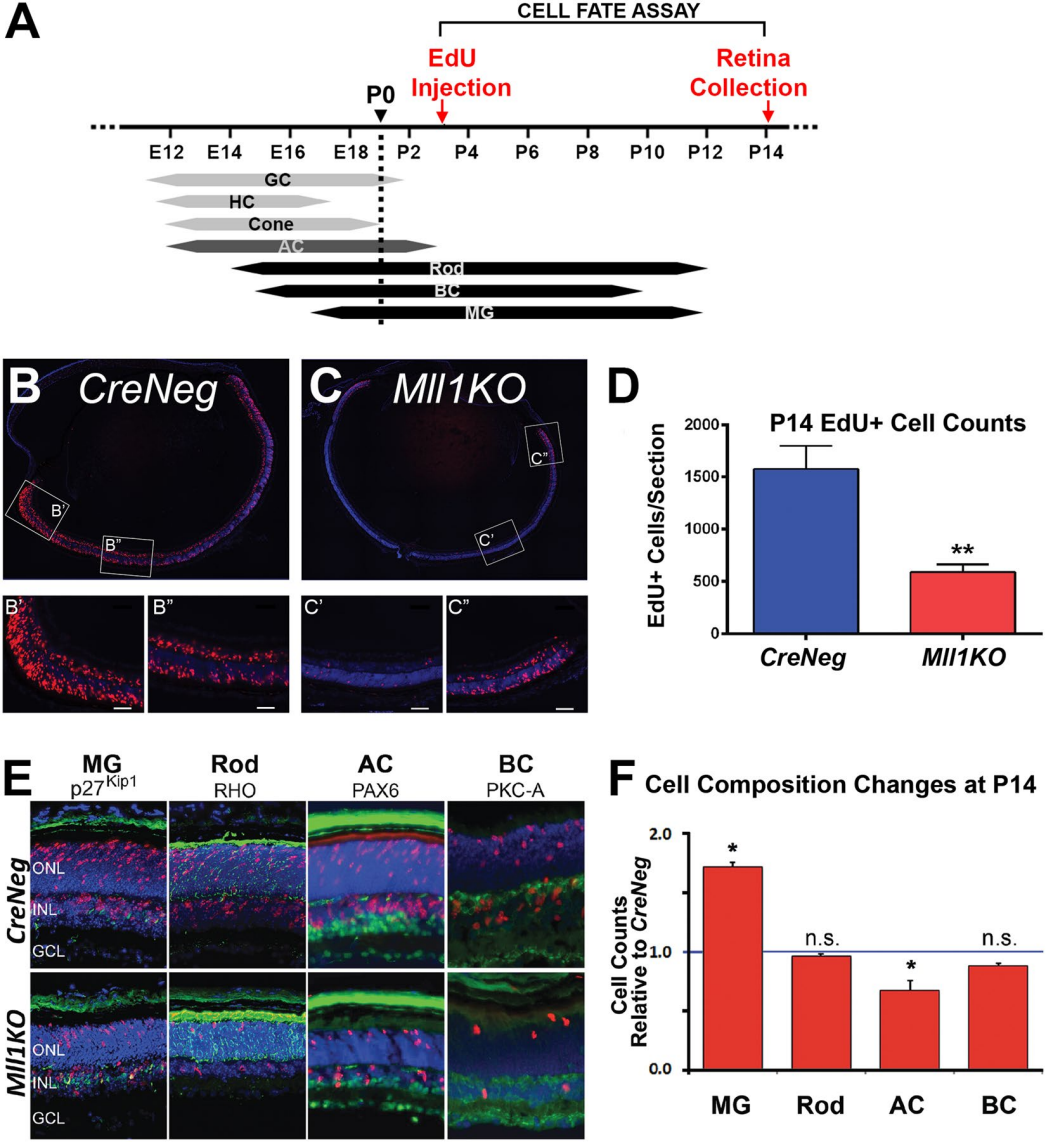
*Mll1* deficiency alters neurogenic vs gliogenic capacity of late progenitor cells. **(A)** Schematic diagram showing experimental strategy for labeling late-born cells: P3 mouse pups were IP injected with EdU. At P14 retinas were collected to count numbers of four late-born cell types. (**B**,**C)** Distribution of EdU positive cells (red) in DAPI-labeled *CreNeg* (**B**) and *Mll1KO* (**C**) P14 retina cross-sections. Inserts (B’,B”,C’,C”) show details at higher magnification of the areas indicated. (**D**) Quantification of EdU positive cells across entire retinal cross-sections, three sections per sample and three samples per genotype. Results are represented as mean + SEM. Two-way ANOVA, **p < 0.0001. (**E**) Retina sections labeled for EdU (red) were co-stained with antibodies for cell fate markers (green) as indicated. (**F**) Cell counts for the different cell types derived from P3 EdU-labeled progenitor cells in *Mll1KO* retinas (red bars), normalized to counts from *CreNeg* littermates. Error bars represent SEM from three biological replicates (n = 3). *p < 0.05, n.s. = not significant by two-way ANOVA with Sidak’s multiple comparisons.

Next, we examined the cell types generated from late RPCs using EdU pulse-chase. EdU-labeled P3 retinal progenitors were assessed for their fate choices at P14, when retinal cell type specification is complete^5^ (Fig. 6A). P3 progenitors give rise to Rod, BC and a few AC neurons, and Muller glia^5^. As expected, cross-sections of P14 *CreNeg* retinas showed many EdU+ cells in both the ONL and INL with a periphery-to-central gradient (Fig. 6B,B’,B”). In contrast, *Mll1KO* retinas had fewer EdU+ cells, mostly seen in the far peripheral regions (Fig. 6C,C’,C”). Quantification revealed approximately one-third as many EdU+ cells in *Mll1KO* as *CreNeg* retinas (Fig. 6D), consistent with the decreased proliferation described above.

We then co-immunostained retinal cross-sections for four late-born cell types expected from the EdU-labeled cells (Fig. 6E): MG (p27^Kip1^), Rod (RHO), AC (PAX6) and Rod ON-BC (PKC-A). Quantification of double-positive cells (Fig. 6F) revealed that the number of EdU-labeled MG per *Mll1KO* retinal section was almost twice the mean number in *CreNeg* sections, while the number of ACs was reduced by 20%. The proportion of rods and BCs were also slightly but not significantly reduced in the mutant retinas. Thus, P3 progenitor cells in *Mll1*-deficient retinas over-produced glial cells at the expense of neurons, especially AC, suggesting a role of MLL1 in establishing the appropriate cell composition and neuron-to-glia ratio during retinogenesis.

### RNAseq analysis supports complex changes of retinal cell composition

**Figure 7:**
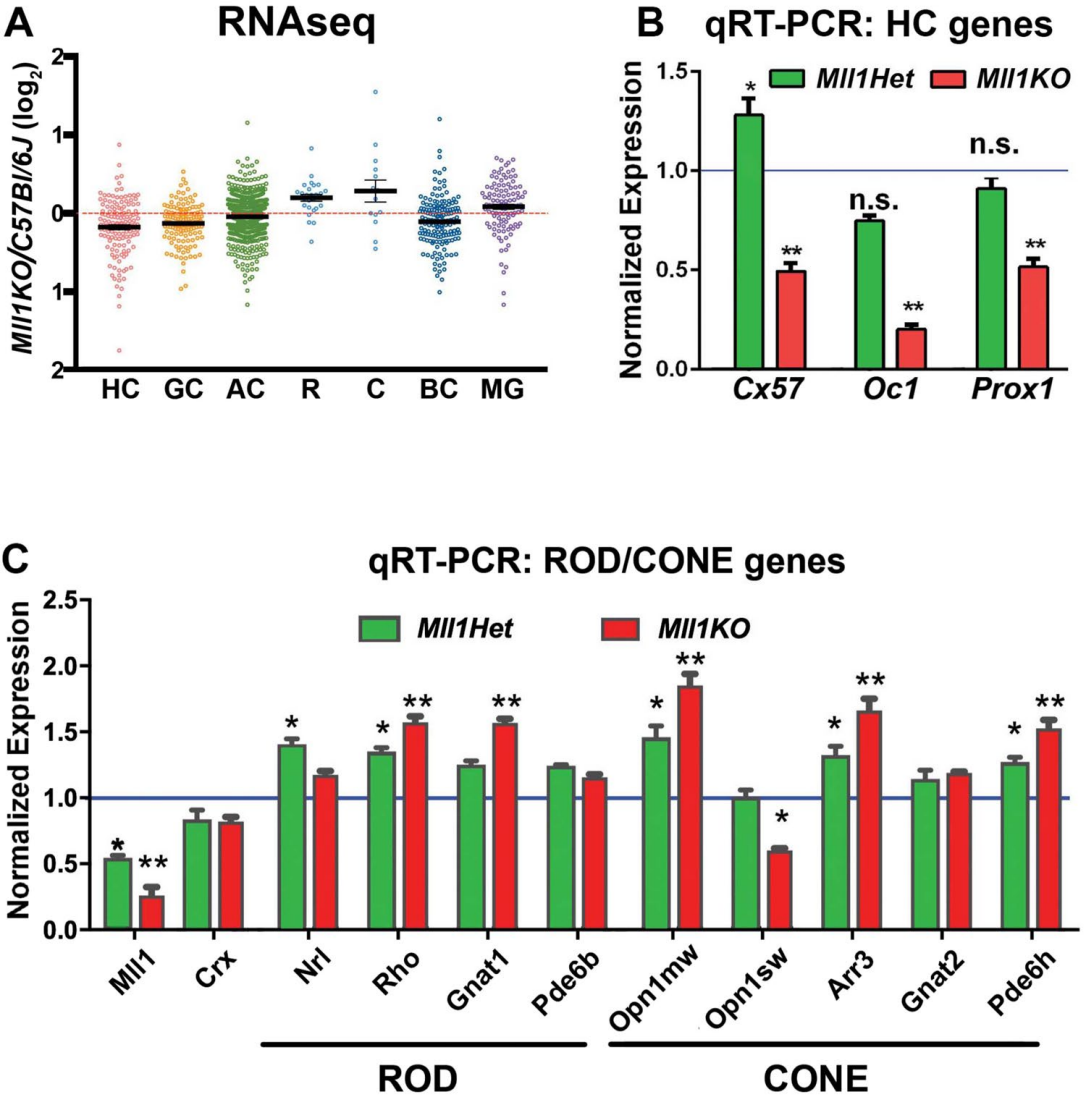
MLL1 is required for appropriate cell-type-specific gene expression. (**A**) RNAseq analysis at P14 (plotted as the ratio of transcripts in *Mll1KO* vs *C57Bl/6J* samples) shows over-representation of Rod (R), Cone (C) and glial (MG) genes and reduction of inner-retina neuron genes in *Mll1*-deficient retinas. (**B**) Quantitative (q)RT-PCR verification of selected horizontal gene expression at P14 in *Mll1Het* and *Mll1KO* retinas relative to *CreNeg* littermates. Primer sequences are given in Supplementary Table S3. (**C**) qRT-PCR verification of selected rod and cone gene expression at P14. Results are presented as fold-expression relative to *CreNeg* samples + SEM (n ≥ 3). *p < 0.05, **p < 0.0001 compared to *CreNeg* by two-way ANOVA with Tukey’s multiple comparisons.

To determine if the altered cell composition correlated with gene expression changes in specific retinal cell types (subtypes), RNAseq analysis was performed on triplicate retinal samples of *Mll1KO* (*KO*) and *wild-type* (*WT*) control mice at P14. Spearman correlation coefficients of RNAseq datasets (Supplementary Fig. S4) showed that replicates within each genotype group clustered together, and the *KO* samples were clearly distinct from the *WT* samples, indicating altered gene expression in *KO* retinas. Genes representing different cell types were grouped according to previously-published datasets defined by retinal single-cell drop-seq^43^, and plotted according to expression differences between *KO* vs. *WT*. Figure 7A shows expression level trends of genes expressed in six neuronal and Muller glial cell types. Overall, four gene groups representing horizontal, ganglion, amacrine and bipolar neurons were decreased in *Mll1KO*, while genes transcribed by rod/cone photoreceptors and Muller glia cells were expressed higher in *Mll1KO* than *WT*. Further analyses for AC and BC subtypes (Supplementary Fig. S5A) showed decreased gene expression in all BC subtypes in *Mll1KO*, while gene expression levels varied in different AC subtypes, with late-born AC subtypes showing greater reductions than earlier-born subtypes (Supplementary Fig. S5B)^43^. Overall, RNAseq results support cell composition changes and altered neuron-to-glia ratio from the late RPCs in *Mll1KO* retina (Figs 5 and 6).

To validate RNAseq results, we performed qRT-PCR on selected genes for the severely reduced HC set (Fig. 7B) and moderately increased rod/cone sets (Fig. 7C). Consistent with RNAseq results, three HC-specific genes, *Cx57*, *Oc1* and *Prox1*, were all expressed at lower levels in P14 *Mll1KO* retinas than *CreNeg* controls (Fig. 7B). *Oc1* and *Prox1*, which encode two TFs essential for HC fate specification^14,15,44^, were reduced by 80% and 50%, respectively, in P14 *Mll1KO*. In contrast, rod/cone genes were generally expressed at similar or relatively higher levels in both *Mll1Het* and *Mll1KO* compared to the controls (Fig. 7C). Since the ONL thickness of *Mll1KO* retinas was relatively unaffected (Supplementary Fig. S1M), increased rod/cone gene expression likely reflects the decrease in other cell types that contribute to total retinal RNA, as a result of preferential inner neuron reductions (Supplementary Fig. S1N).

### Histone modifications consistent with RNAseq analysis

In the RNAseq analysis and histological examinations, we were surprised to see that the rod photoreceptors, which make up the majority of retinal cells, look relatively normal. As MLL1 is known to activate cell type-specific gene expression by methylation of lysine 4 of histone H3 (H3K4), we wondered how levels of these histone marks are affected in mutants. Whole retinal extracts displayed no differences in levels of H3K4me3 or the repressive mark H3K27me3 in *Mll1KO* vs. *CreNeg* control mice (Supplementary Fig. S6A,B). Furthermore, immunostaining for several modified histones, including the active marks H3K4me2, H3K4me3 and H3 Pan-Ac and the repressive mark H3K27me3, showed no gross differences between cell types within *Mll1KO* retinas or when compared to *CreNeg* control samples (Supplementary Fig. S6C). Together, these results suggest that there must be other mechanisms to compensate for the loss of MLL1 in guiding development and maintenance of photoreceptors and some other retinal cell types.

### *Mll1* deficiency disrupts the integrity of horizontal cells

**Figure 8:**
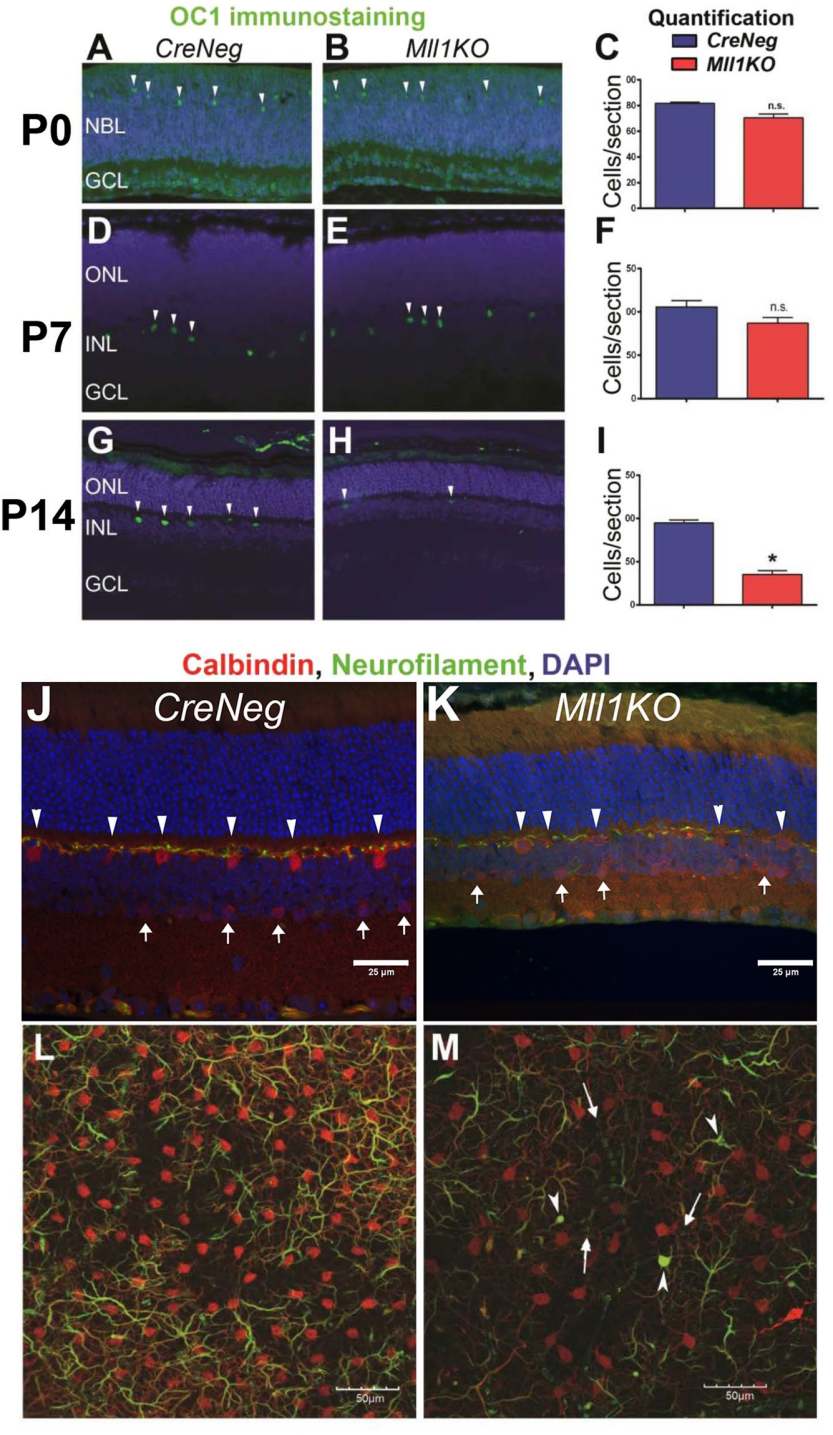
MLL1 is required for horizontal cell integrity. Retinal cross-sections of *CreNeg* and *Mll1KO* mice at P0 (**A**,**B**), P7 (**D**,**E**) and P14 (**G**,**H**), immunostained for OC1 (green) to mark horizontal cells (arrowheads). Quantification of OC1 + cells per retina cross-section at P0 (**C**), P7 (**F**) and P14 (**I**), presented as mean OC1 + cells per cross-section + SEM. *p < 0.05, n.s. = not significant by two-way ANOVA with Tukey’s multiple comparisons (n = 3). To further assess horizontal cell distribution and connectivity in 1MO retinas, immunostaining was performed on retinal cross-sections (**J**,**K**) for Calbindin (red), which labels cell bodies of HC and AC, and Neurofilament medium chain (green), which labels their axons. (**J**,**K**) Retinal cross-sections show the clear spatial distribution of HC (arrowheads) and AC (white arrows). Flat mounts of 1MO *CreNeg* (**L**) and *Mll1KO* retinas (**M**), imaged with confocal microscopy at the level of the OPL and outer INL revealed defects in the HC mosaic and axon distribution network, as well as interrupted neurofilaments (arrows) and axon varicosities (arrowheads) in *Mll1KO* retinas. Antibody information is given in Supplementary Table S2.

Although horizontal cells (HC) are an early-born cell type, their gene expression profile was one of the most severely affected in *Mll1KO* retinas. To determine whether this change resulted from altered cell fate specification or defective cell maintenance, we performed immunostaining at three postnatal ages for OC1 (Fig. 8A–I), a transcription factor essential for HC fate specification and survival^14,44^. At P0, both *Mll1KO* and *CreNeg* control retinas showed comparable numbers and locations of OC1 + HCs (Fig. 8A–C). Thus, initial HC specification and migration appeared normal in *Mll1KO* retinas. At P7, OC1 + HCs were largely maintained in *Mll1KO* retinas (Fig. 8D–F). However, by P14, the number of OC1 + HCs in *Mll1KO* retinas dropped significantly to less than 40% of the controls (Fig. 8G–I). The OC1 staining intensity was also dramatically reduced in the remaining HCs compared to those in the control retinas (Fig. 8H vs G), suggesting that *Mll1KO* HCs lose their integrity and/or die between P7 and P14.

To confirm reductions in HCs and reveal changes in the HC mosaic and neurite distribution, we performed co-immunostaining for Calbindin and Neurofilament (NF) on 1MO retina sections and flat-mounts. While antibodies to NF label HC axons, Calbindin marks HC and AC somata and dendrites in retina cross sections^45^. In *CreNeg* retinas, intensely stained Calbindin-positive HC somata are positioned at the border between INL and OPL, while lightly-stained Calbindin-positive AC somata are located in the inner INL and GCL (Fig. 8J). Compared to the controls, *Mll1KO* retinas had fewer and fainter Calbindin-positive HCs (Fig. 8K, white arrowheads) and ACs (Fig. 8K, white arrows). In addition, the mutant OPL appeared thinner and the IPL laminae more disorganized in *Mll1KO* retinal sections at 1MO. Next, we performed confocal microcopy to visualize the HC plane on flat-mounted retinas co-stained for Calbindin (red) and NF (green). In *Mll1KO* retinas, Calbindin + HC somata and dendrites were sparser and fainter than in *CreNeg* controls (Fig. 8M vs L). Many HC axons in *Mll1KO* retina were short and abnormal in appearance, some retracted to the cell body or axonal swellings (Fig. 8M, white arrowheads). These defects could lead to deficits in the receptive field and abnormal visual function. Collectively, these results suggest that MLL1 is not required for initial HC fate specification, but is indispensable for maintaining HC integrity, including identity, gene expression and axon network.

### *Mll1KO* retinas fail to develop normal OPL synapses

**Figure 9:**
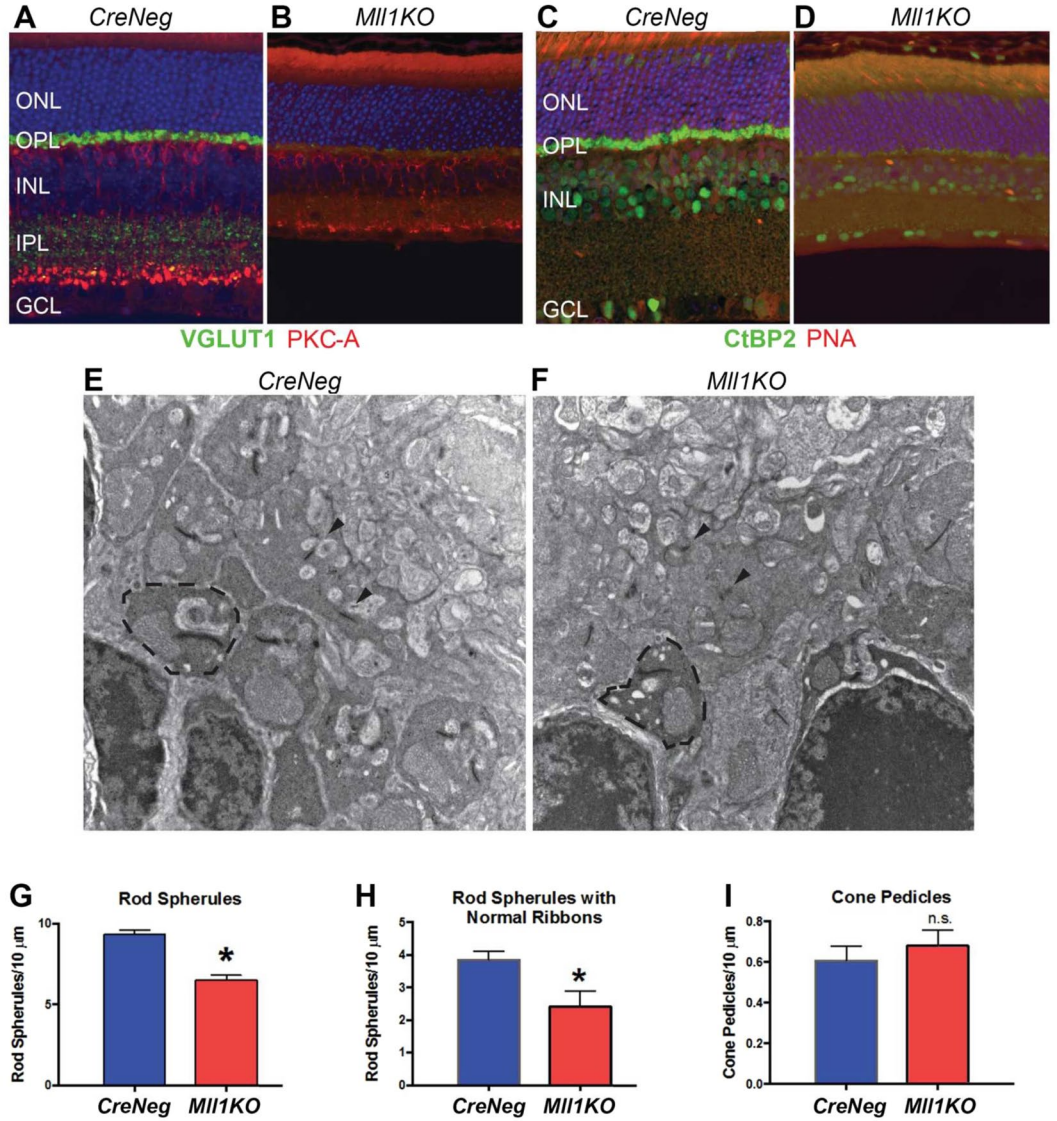
*Mll1KO* retinas have synaptic defects. Immunostaining of 1-month retina cross-sections for synaptic marker VGLUT1 (green) and bipolar marker PKC-A (red) (**A**,**B**), or for CtBP2 (Ribeye, green) and peanut agglutinin (PNA, red) for cones (**C**,**D**) reveal markedly reduced photoreceptor presynaptic terminals in the *Mll1KO* outer plexiform layer (OPL). Antibody information is given in Supplementary Table S2. In electron microscopy images of the OPL (**E**,**F**), representative rod spherules are marked with dotted lines and synaptic ribbons indicated by arrowheads. Quantification of the total number of rod spherules (**G**) and the number of spherules with normal ribbons (**H**), displayed as mean rod spherules per 10μm region + SEM. Quantification of the number of cone pedicles (**I**), displayed as mean cone pedicles per 10μm + SEM. *p < 0.05, n.s. = not significant by two-way ANOVA (n ≥ 4).

To determine if the visual signal transmission defects detected by ERG analysis can be explained by changes in OPL structural integrity, we performed immunostaining for two presynaptic proteins expressed in photoreceptors: VGLUT1 is a glutamate transporter necessary for loading glutamate into synaptic vesicles and for photoreceptor synaptic transmission^46,47^. CtBP2 (Ribeye) is an essential structural component of pre-synaptic ribbons^48^. Both markers were clearly visible in the OPL of 1MO *CreNeg* mice but markedly decreased in age-matched *Mll1KO* mice (Fig. 9A–D), indicating abnormalities in the photoreceptor pre-synaptic terminals. Next, we examined ultrastructural changes in OPL synapses using transmission electron microscopy (TEM). Randomized TEM micrographs from both control and mutant samples (Fig. 9E,F) were analyzed for rod spherules and cone pedicles. *Mll1KO* retinas had fewer rod spherules than *CreNeg* littermate controls (Fig. 9G), and fewer of those showed normal shaped ribbons in *Mll1KO* than in controls (Fig. 9H). No significant difference in the number of cone pedicles was detected between mutants and controls (Fig. 9I). These findings suggest that MLL1 is required for establishing normal photoreceptor synapses for visual signal transmission.

## Discussion

Conditional knockout of MLL1 in developing retinas resulted in significant reductions of retina thickness, detected as early as P0. This phenotype likely results from a reduction of the progenitor cell pool, particularly of late RPCs, but not massive cell death. First, postnatal proliferating cell numbers were reduced by both Ki67 immunostaining and EdU incorporation. Second, cell reductions were detected across many neuronal cell types by immunostaining and RNAseq analyses. Third, apoptotic cells were not increased in mutant retinas at any ages tested. However, due to the transient and limited nature of the assays performed, we cannot rule out the possibility that altered cell death might contribute to the reduction of specific neurons. Both reduced proliferation and increased apoptosis were detected in *Mll1*-deficient hematopoietic stem cells (HSCs)^49^.

The shrinkage of the proliferating cell pool in *Mll1KO* retina is likely due to abnormal cell cycle progression. P0 *Mll1KO* retinas had no significant change in the proportion of proliferating cells in S-phase (EdU+), but did display an increased percentage of M-phase cells (PH3+) compared to *CreNeg* controls (Fig. 4), implicating altered cell cycle progression, possibly at M-phase exit. This can be further verified by flow cytometry analyses to quantify labeled cells in different cell cycle phases. Future clonal analyses^50,51^ will determine the impact of cell cycle deficits on the capacity of each progenitor to produce designated cell types. Overall, however, our findings are consistent with published reports demonstrating that MLL, the human orthologue of MLL1, is required for both S-phase entry and M-phase progression in cultured human cancer cell lines, acting at the G1/S and G2/M transitions^38^. Although the molecular differences between normal and *Mll1*-deficient RPCs remain to be determined, similar RPC proliferation deficits were also seen in vertebrate retinas lacking several other histone modifying enzymes, particularly those involving the repressive histone mark H3K27me3^52–55^. This suggests the importance of homeostasis of histone modifying enzymes in retinogenesis.

The EdU pulse-chase experiments showed that *Mll1*-deficient postnatal retinal progenitor cells have reduced neurogenic but not gliogenic potential. RNAseq analyses in *Mll1KO* retinas showed that gene sets specific to individual neuronal cell types (and subtypes) were widely affected, but the gene set for Muller glia was minimally affected. These results are consistent with previous findings from mouse brain, where *Mll1*-deficent neural stem cells in the subventricular zone were able to survive and differentiate into glia, but their capacity to differentiate into neurons was significantly diminished^33^. *Mll1* was also required for the expression of neurogenic but not gliogenic transcriptional modules^56^. Although these findings suggest a common mechanism by which *Mll1* deficiency alters the neurogenic potential of retina progenitors, that mechanism remains to be determined.

Importantly, our cell biology assays and whole retina RNAseq analyses informed by recently-published single-cell RNAseq datasets have revealed that different neuronal subtypes in the retina show distinct sensitivities to *Mll1* deficiency: the inner neurons, particularly HCs and some of the late-born ACs, are highly sensitive, while rods/cones in the ONL are less vulnerable. *Mll1* transcript levels in normal mouse retinas are relatively higher in INL and GC layers than the ONL, which may provide one explanation for the severe inner retina phenotype in *Mll1KO*. Finally, analysis of bulk retina RNAseq results in accordance with published single-cell datasets allowed us to decipher cell-type-specific changes in both major and minor cell populations.

Our study also identified a specific role for MLL1 in HC integrity and survival, which is essential for synaptic layer organization and retinal function^14,45,57,58^. In *Mll1KO*, HCs were initially born, specified and had migrated to the correct position by P7. However, by P14, *Mll1*-deficient HCs lost immunostaining for the key HC transcription factor OC1 (Fig. 8A–I), and showed a sparse and uneven distribution of their neurite network (Fig. 8M). Gene expression analyses showed that loss of HC-specific markers largely occurred at the transcriptional level, and can be explained by the loss of transcripts for essential transcription factors OC1 and PROX1 in the HC lineage (Fig. 7B). The mechanism for this loss remains to be determined. HC epigenetic maturation could require MLL1, but this is technically challenging to test, as HCs represent less than 0.6% of all retinal cells^43^. Similarly, other post-neurogenesis abnormalities may also affect the maintenance of other early-born neurons, as GCs and early-born ACs were modestly reduced in *Mll1KO* at P24, but not at P0 (Fig. 5).

MLL1 appears not to be required for rod/cone specification and survival, but decreased ERG waves in *Mll1KO* mice imply defective phototransduction in both cell types. More severely affected B-waves than A waves in dark-adapted ERGs suggest abnormalities in visual signal transmission from photoreceptors to the inner retina. Supporting this, *Mll1KO* photoreceptor synaptic terminals lack the ribbon synaptic marker Ribeye (CtBP2) and glutamate transporter VGLUT1. Several pieces of evidence suggest these deficits are secondary to the loss of post-synaptic interneurons or terminal structures. First, RNAseq and qRT-PCR results suggest that rod/cone gene transcription in *Mll1KO* was largely normal, including those genes coding for the missing presynaptic markers *Slc17a7* (coding for VGLUT1) and *Ctbp2* (coding for Ribeye). Thus, the presynaptic terminal abnormalities likely stem from a post-transcriptional problem, such as mis-assembly of ribbon synapses. Second, conditional knockout of *Mll1* in rods (using *Rho-iCre75*) or cones (using *HRGP*-*Cre*) did not yield apparent changes in retinal structure or function up to 6 months of age (data not shown), suggesting that, in rods and cones, MLL1 is dispensable for cell differentiation and maintenance as well as for cell survival. Third, development of appropriate presynaptic terminals depends on the integrity of postsynaptic neurons, particularly HCs. Loss of HCs during retinal development causes defects in retinal layer structure and photoreceptor ribbon synapses^14,45,57,58^. In our study, *Mll1KO* retinas began to lose HCs at P7-P14 (Fig. 8A–I), a critical period for establishing pre-synaptic and post-synaptic connections in the normal retina^59^. Ablating all HCs in adult retinas also produced severe presynaptic structural deficits, post-synaptic remodeling at the OPL and photoreceptor degeneration, particularly affecting the rods^60^. Conditional knockout of *Mll1* in HCs (e.g. using *Cx57-Cre*) will further clarify the cell non-autonomous effects of *Mll1-* deficient HCs. In addition, dysfunction/loss of other inner retina neuronal types such as BCs and ACs, or their remodeling after HC loss, could also contribute to abnormal synapses and visual function impairment in *Mll1KO* retinas, in either cell autonomous or non-autonomous ways.

Our results provide new insight into a cell type-specific role of MLL1, which is widely expressed in many tissues and cell types. The retina expresses three other members of the MLL family: MLL2 (KMT2b), MLL3 (KMT2C) and MLL4 (KMT2D). *In situ* hybridization and qRT-PCR analyses showed that their transcripts followed similar spatial and temporal expression patterns as *Mll1* (data not shown). However, unlike *Mll1*, single conditional knockout of *Mll2* or *Mll3* using *Chx10-Cre* did not yield significant structural or functional changes in the retina (Brightman and Chen, unpublished results). Furthermore, RNAseq and qRT-PCR analyses of *Mll1KO* retinas showed that *Mll2* and *Mll3* transcripts were expressed at similar levels as in normal retina. These results support a non-redundant role of MLL1 in generating and maintaining specific retinal neuron types (subtypes). Future double conditional knockout studies will shed light on the overlapping and redundant roles of MLLs in the retina.

**Figure 10:**
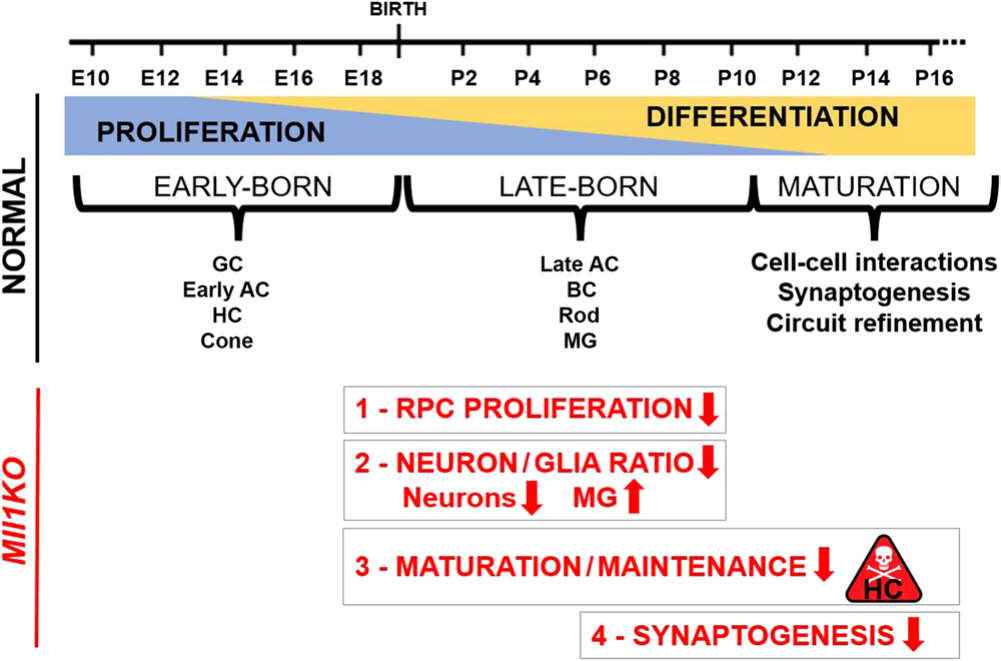
Summary of normal retinal development (black text) and the impact of *Mll1*-deficiency (in red).

In conclusion, using a loss-of-function approach, we have found that MLL1 plays specific roles in retinal development at multiple levels as illustrated in Fig. 10: (1) RPC proliferation and cell cycle progression, (2) neuronal cell type composition and neuron-to-glia ratio, (3) horizontal cell maintenance, and 4) organization of synaptic connections for visual function. Our study provides new insight into how a general histone modifying enzyme contributes to the genesis and terminal differentiation of a CNS tissue in addition to its known roles in cancer and stem cells. MLL1 function has been implicated in learning and memory^31,34,35^. Our study emphasizes a complex mechanism for making appropriate synaptic connections between pre- and post-synaptic neurons, which may shed light on MLL’s role in neuronal signal transmission, processing and synaptic plasticity.

Finally, *Mll1KO* affected retinal cell types significantly differ from those conditional knockouts of other histone modifying enzymes. Deficiency of the enzymes regulating the repressive mark H3K27me3, such as EED and EZH2 in the polycomb repressive complex 2 (RPC2), causes deficits in BCs, Rods and MGs^52,61^. Together, these findings emphasize the need to elucidate overlapping and unique roles of individual histone modifying enzymes and their homeostasis in the development and maintenance of particular cell types/tissues.

## Methods

### Mouse lines and PCR genotyping

All mice used in this study were backcrossed to *C57BL/6 J* mice (the Jackson Laboratory, Bar Harbor, Maine; Stock # 000664) and housed in Washington University School of Medicine barrier facilities. *Mll1 flox* mice^29^ were obtained from Patricia Ernst at University of Colorado, Denver. *Chx10-Cre*^37^ mice were provided by Connie Cepko at Harvard University. Both females and males were included in each experiment.

For genotyping, genomic DNA was isolated from mouse tail clips using the Gentra Puregene Tissue Kit (Qiagen, Germantown, MD). PCR amplification was performed using Jumpstart RedTaq (Sigma-Aldrich, St. Louis, MO) and PCR primers as published for each mouse.

All animal procedures were conducted according to the Guide for the Care and Use of Laboratory Animals of the National Institute of Health, and were approved by the Washington University in St. Louis Institutional Animal Care and Use Committee.

### Electroretinogram

Whole animal ERGs were performed on 1, 3, or 6-month old mice and littermate controls using a UTAS-E3000 Visual Electrodiagnostic System with EM for Windows (LKC Technologies, Inc., Gaithersburg, MD). Mice were dark-adapted overnight. Prior to testing mice were anesthetized with 80 mg/kg ketamine and 15 mg/kg xylazine under dim red-light illumination. Mouse body temperature was kept at 37 ± 0.5 °C with a heating pad controlled by a rectal thermistor probe (FHC Inc., Bowdin, ME). Pupils were dilated with 1% atropine sulfate solution (Bausch and Lomb, Tampa, FL) and 1.25% hydroxypropyl methylcellulose (GONAK; Akorn Inc., Buffalo Grove, IL) was placed on the eyes. Platinum 2.0 mm loop electrodes were carefully placed on the cornea of each eye. A ground electrode was placed under the skin of the mouse’s back and a reference electrode was inserted under the skin of the mouse’s head. Retinal response to brief (10 μs) full-field light flashes of increasing intensity were recorded; maximum flash intensity for dark-adapted testing was 0.895 cd*s/m^2^. Following dark adapted tests, mice were light-adapted in dim light (29.2 cd/mm) for 10 minutes and exposed to 10 μs light flashes of increasing intensity; maximum flash intensity for light-adapted testing was 2.672 cd*s/m^2^. Responses from several trials were recorded and averaged. The mean peak amplitudes of dark-adapted A waves and B waves and light-adapted B waves were graphed against log light intensity (cd*s/m^2^). At each age responses were averaged by genotype and compared using two-way ANOVA with multiple comparisons (Sidak’s for 2 genotypes or Tukey’s for 3 or more genotypes).

### Histology, Immunohistochemistry and EdU Labeling

Eyes were enucleated at various time points, dissected with a corneal tag marking the superior side of the retina, and fixed in 4% paraformaldehyde at 4 °C overnight for paraffin embedded sections. For frozen sections, the eyes were fixed for 30 min only, washed in PBS for 10 min, incubated in 30% sucrose overnight, embedded in OCT and frozen on dry ice for cryosectioning. Frozen sagittal cross-sections were cut 7 microns thick and placed on poly-D lysine coated slides (Thermo Fisher Scientific, Waltham, MA). Paraffin embedded eyes were cut 5 microns thick on a microtome, stained with Hematoxylin and Eosin to examine retinal morphology. For immunostaining on paraffin embedded retinal sections, antigen retrieval was performed for all sections and blocked in 1% BSA/0.1% Triton-x-100 in 1x PBS. Frozen sections were blocked with 5% nonfat dry milk, 2% BSA and 10% normal goat serum in PBS. See Supplementary Table S2 for list of primary antibodies. Secondary antibodies were applied 1:400 Goat anti-rabbit, anti-mouse or anti-guinea pig antibodies coupled to Alexa Fluor 488, Alexa Fluor 568 or Alexa 647 (Invitrogen Molecular Probes, Thermo Fisher Scientific). All slides were counterstained with hard set mounting medium with DAPI (Vectashield, Vector Laboratories, Inc, Burlingame, CA). Slides were imaged on an Olympus BX51 microscope or Leica DB5500 microscope.

For EdU Labeling: Mice were injected IP with 10uL of 10 mM EdU/per gram mouse weight. At harvest, eyes were dissected and fixed as described above. EdU labeling was done using the Click-iT EdU Assay Kit (Invitrogen, Thermo Fisher Scientific).

Immunohistochemistry on whole flat-mounted retinas was performed according to Soto *et al*.^45^: After dissection, the retina was placed flat on a piece of filter paper, GCL side up, then fixed in 4% paraformaldehyde for 30 min, cryoprotected in a sucrose gradient overnight. Retinal flat mounts were blocked for 4 hours, incubated for 5 days at 4 °C, and incubated in secondary antibody solution for 2 days at 4 °C. Flat mounts were imaged on an Olympus FV1000 confocal microscope.

### Morphometry and Cell Count Analysis

For morphometry, 20x images of hematoxylin and eosin stained retinal cross-sections were stitched together. The thickness of retinal layers was measured using Image J software (imagej.nih.gov/ij). Thicknesses were measured at regular distances from the optic nerve head. Results are displayed in a spider graph. Measurements were done blinded to genotype and sample number. For cell type quantification, a minimum of 3 animals were collected for each genotype and 3 sections per eye were stained and imaged. All counting was done blinded. Statistical analyses were performed using two-way ANOVA with multiple comparisons (Sidak’s for 2 genotypes or Tukey’s for 3 or more genotypes). p < 0.05 CI: 95%. All statistical analysis was performed using Graphpad Prism 6.0 (GraphPad Software, La Jolla, CA).

### Transmission Electron Microscopy (TEM)

For TEM, mice were anesthetized with ketamine (80 mg/kg) and xylazine (15 mg/kg) and perfused with 2% paraformaldehyde/2.5% glutaraldehyde in 0.1 M sodium phosphate buffer. Retinas were fixed in 2% paraformaldehyde/3% glutaraldehyde in 0.1 M phosphate buffer for 24 hours. They were then incubated in 1% osmium tetroxide for 1 hour and stained with 1% uranyl acetate buffer for 1 hour. Blocks were dehydrated in an acetone gradient and embedded. Semi-thin sections were stained with toluidine blue, uranyl acetate and lead citrate and imaged on a Hitachi H7500 electron microscope. 4 *CreNeg* and 5 *Mll1KO* retinas were collected. 25 images at 15000x magnification were randomly sampled per animal blinded to genotype. Rod spherules, cone pedicles and rod spherule ribbon shapes were counted blinded using Image J software. Comparison between genotypes was done by ordinary two-way ANOVA with Sidak’s Multiple Comparisons using Graphpad Prism 6. p < 0.05 CI:95%.

### *In situ* hybridization

*In situ* hybridization was performed on paraffin embedded retinal sections using the QuantiGene ViewRNA ISH Tissue Assay Kit (Affymetrix, Thermo Fisher Scientific) with a custom designed *Mll1* probe (Fig. 1A). Slides were co-stained with DAPI and imaged.

### qRT-PCR

RNA was purified from retinas from 2 mice for each sample using the Perfect Pure RNA Tissue Kit (5 Prime, Thermo Fisher Scientific) and quantified using a NanoDrop ND-1000 (Nanodrop, Thermo Fisher Scientific). cDNA was synthesized from 1 μg of RNA with Roche Transcriptor First Strand cDNA Synthesis Kit (Roche Diagnostics Corporation, Indianapolis, IN). Primer sequences not listed in Supplementary Tables have been previously published^62^. EvaGreen with Lox Rox (Bio-Rad Laboratories, Hercules, CA) was used as the reaction mixture along with 1 μM primer mix and diluted cDNA in triplicate for each sample. Samples were run using a two-step 40 cycle amplification protocol on a Bio-Rad CFX96 Thermal Cycler (Bio-Rad Laboratories, Hercules, CA). Primers were designed using MacVector software (MacVector, Apex, NC) and synthesized by Integrated DNA Technologies (IDT, Coralville, Iowa). During design primers were tested to ensure efficiency between 90 and 110% and specificity. Cqs were analyzed using QBase qPCR analysis software (https://www.qbaseplus.com; Biogazelle, Belgium). Reference genes were verified using GeNorm analysis in QBase software. Gene expression was normalized to reference genes in QBase. Expression in different genotypes was compared using ordinary two-way ANOVA. p < 0.05 CI:95% Statistical analysis was done in Graphpad Prism 6.

### RNA-seq library preparation, sequencing and data analysis

To prepare RNAseq libraries, RNA was purified from three biological replicates (2 retinas per sample) using the Perfect Pure RNA Tissue Kit (5 Prime, Thermo Fisher Scientific). cDNA Libraries were prepared as previously described^63^ and sequenced on the Illumina 2500 (1 × 50 bp reads). Data were aligned to *Mus musculus* reference build Ensembl GRCh38.76 using STAR (V2.0.4b) and read counts (CPM) determined with Subread:featureCount (V1.45) and annotated with Ensembl Biomart. The raw datasets are available from Gene Expression Ominibus (GEO https://www.ncbi.nlm.nih.gov/geo/info/linking.html; GSE103264).

Initial data comparison including Spearman Correlation Coefficient (Supplementary Fig. S4) was done in the R environment (V3.4.1; https://www.r-project.org/. To detect differential expression, genes that did not pass the filter criteria of CPM > 5 in at least 5 samples were removed. Filtered count data were compared using default parameters in EdgeR (V3.8.6). Genes defined as informative in discriminating Clusters 1–39 by Macosko^43^, based on Supplementary Table S4; myDiff > 0) were extracted from EdgeR analysis. Supplementary Figure S5 includes the value for all genes in each Cluster list. In Fig. 6A, AC and BC datasets consist of union sets of genes classifying clusters 3–23 and 26–33, respectively, in Supplementary Fig S5 (duplicate values were removed).

### Data availability

The raw and processed RNAseq datasets are available from Gene Expression Ominibus (GEO https://www.ncbi.nlm.nih.gov/geo/info/linking.html; GSE103264).

## Electronic supplementary material

Supplementary Materials
