# Supplementary material for: MLL1 is essential for retinal neurogenesis and horizontal inner neuron integrity
Source: Sci Rep. 2018 Aug 9;8:11902. doi: 10.1038/s41598-018-30355-3 (PMC6085291; doi:10.1038/s41598-018-30355-3)
Supplement: Supplementary file 1 — Supplementary Materials [file 41598_2018_30355_MOESM1_ESM.pdf]

## SUPPLEMENTARY MATERIALS

# MLL1 is essential for retinal neurogenesis and horizontal inner neuron integrity

Diana S. Brightman[1, 3], Rachel L. Grant[5], Philip A. Ruzycki[4], Ray Suzuki[5], Anne K. Hennig[1] and Shiming Chen[1, 2*]

[1]Department of Ophthalmology and Visual Sciences, [2]Department of Developmental Biology, [3]Molecular Cell Biology and [4]Molecular Genetics and Genomics graduate programs, Division of Biology & Biomedical Sciences, [5]College of Arts and Sciences, Washington University, Saint Louis, Missouri, USA

**CONTENTS** **PAGE**

**Figure S1: Development of *Mll1KO* retina.** **2**
**Figure S2: *Mll1KO* retinas do not show increased apoptosis.** **3**
**Figure S3: Proliferation of retinal progenitor cells (RPC) decreases in postnatal *Mll1KO* mice.** **4**
**Figure S4: Hierarchical cluster analysis of RNAseq results.** **5**
**Figure S5: Expression changes of cell type-specific gene sets.** **6**
**Figure S6: Comparison of histone marks in *CreNeg* and *Mll1KO* retinas.** **7**

**Table S1: *Mll1* PCR primer sequences** **8**
**Table S2: Antibodies** **9**
**Table S3: HC gene PCR primer sequences** **10**

**References** **10**

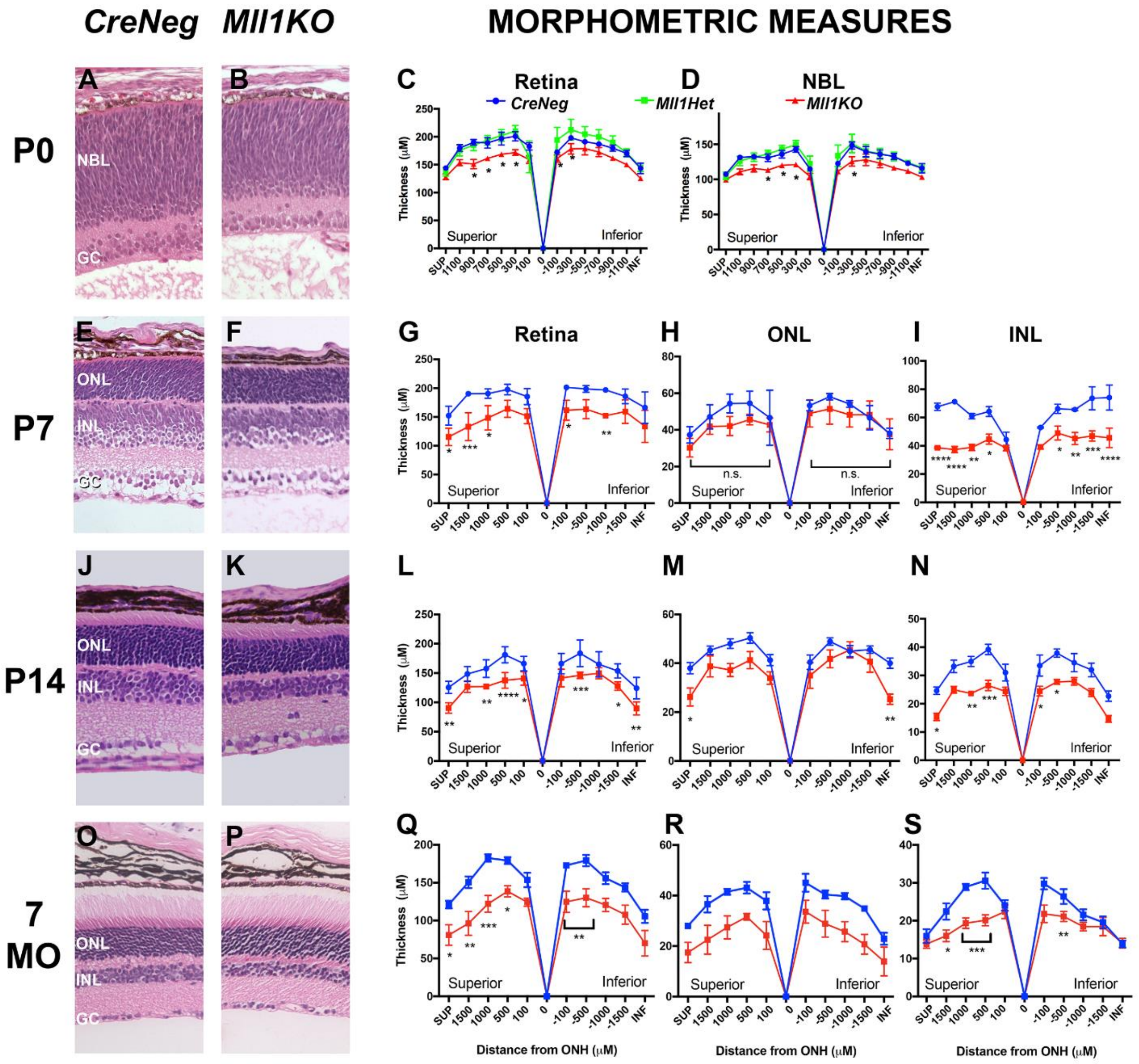


**Supplementary Figure S1:** Development of *Mll1KO* retina. Images of H&E-stained *CreNeg* (**A, E, J, O**) and *Mll1KO* (**B, F, K, P**) retinal cross-sections at the indicated ages, with morphometric measures comparing thickness of the whole retina (**C, G, L, Q**), the NBL (**D**), ONL (**H, M, R**) and INL (**I, N, S**), taken at the indicated positions from the ONH. SUP and INF indicate measurements taken 100um from the superior or inferior edge of the retina. **p*<0.05; ***p*<0.01; ****p*<0.001; *****p*<0.0001, by two-way ANOVA with repeated measures and Sidak's multiple comparisons test (n≥4).

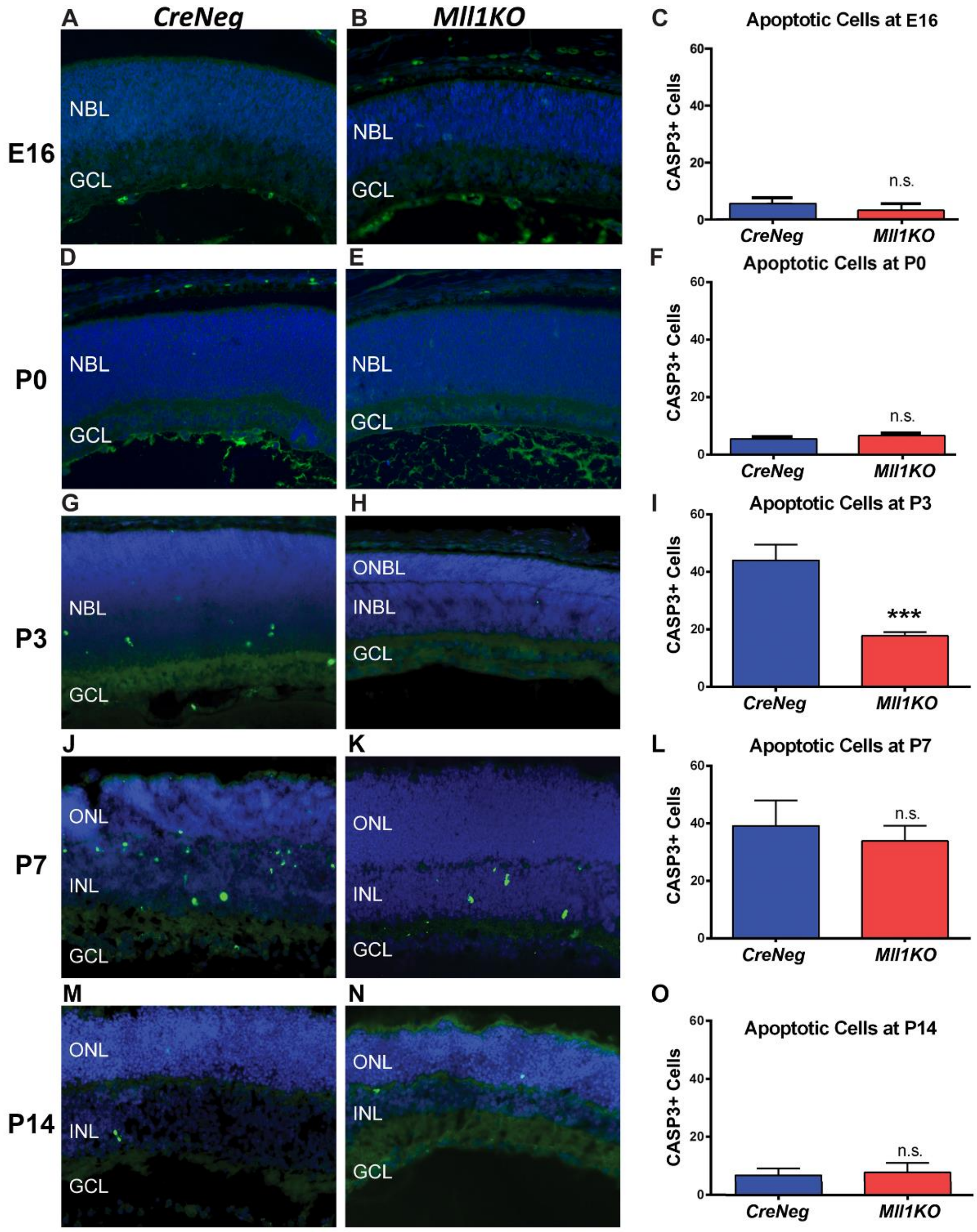


**Supplementary Figure S2:** *Mll1KO* retinas do not show increased apoptosis. Activated Caspase-3 immunostaining (green) with DAPI nuclear stain (blue) of retinal cross sections from *CreNeg* (**A, D, G, J, M**) *and Mll1KO* (**B, E, H, K, N**) mice at the indicated ages. (**C, F, I, L, O**) Quantification of CASP3+ cells per retinal section at each age. Three retinal cross-sections per sample and three samples per genotype were imaged, blinded and counted. The results are shown as mean + SEM. *$p$<0.05, n.s. = not significant by two-way ANOVA (n=3).

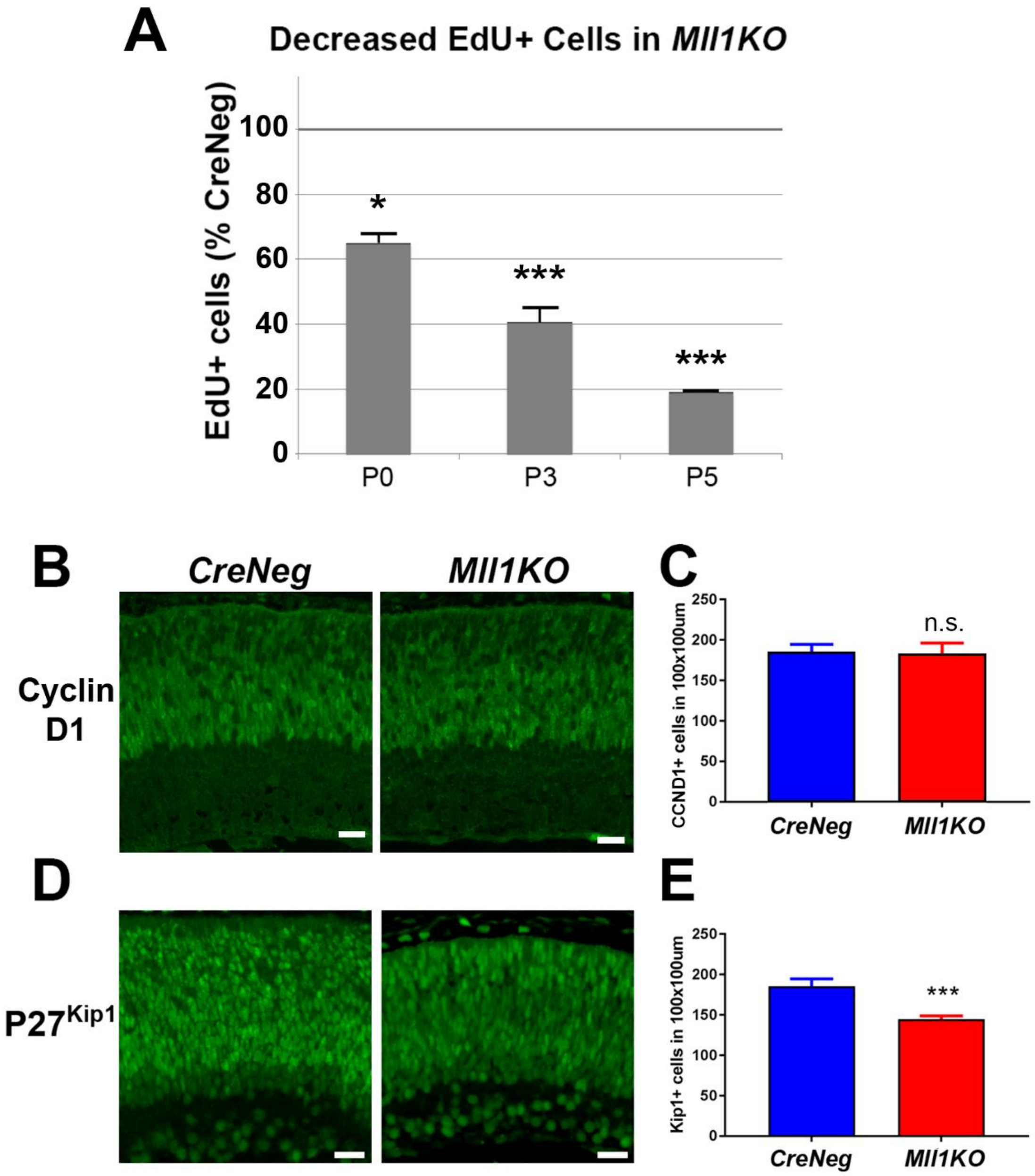


**Supplementary Figure S3:** Proliferation of retinal progenitor cells (RPCs) decreases in postnatal *Mll1KO* mice. (**A**) *Mll1KO* retinas show progressively fewer EdU-labeled cells after a 4-hour pulse than *CreNeg* littermates. Results are shown as mean + SEM. *$p$<0.05; ***$p$<0.0001 by two-way ANOVA with Tukey's multiple comparisons (n=3). (**B**) P0 retinal cross sections of *CreNeg* and *Mll1KO* retinas were immunolabeled with anti-Cyclin D1 (CCND1) (green) for proliferating cells. (**C**) Quantification of labeled cells in 100umX100um areas across each section, shown as mean + SEM. No significant differences (n.s.) were detected between the two genotypes. (**D, E**) Retinal cross-sections from the same mice as in Panel B were immunolabeled for the cyclin-dependent kinase inhibitor p27$^{Kip1}$ and quantified. Scale bar = 20um. ***$p$<0.001 by two-way repeated-measures ANOVA with Sidak's multiple comparisons test (n=3).

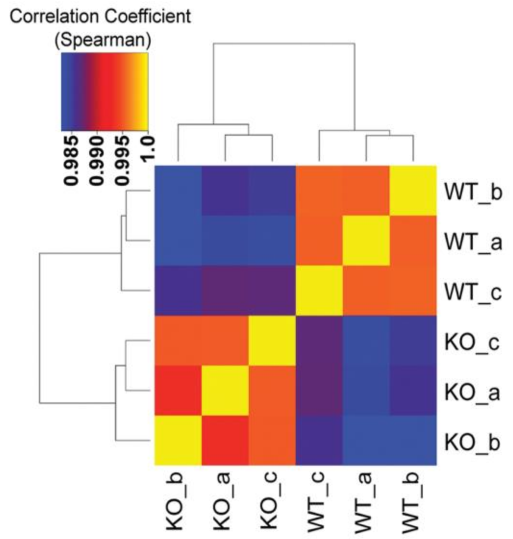


**Supplementary Figure S4:** Hierarchical cluster analysis of RNAseq results (shown as heatmap representing Spearman's Rank Correlation Coefficient) shows a clear distinction between the *Mll1KO* ("KO") and *C57Bl/6J* ("WT") libraries sequenced, as well as reproducibility between the three biological replicate libraries ("a", "b" and "c") representing each genotype.

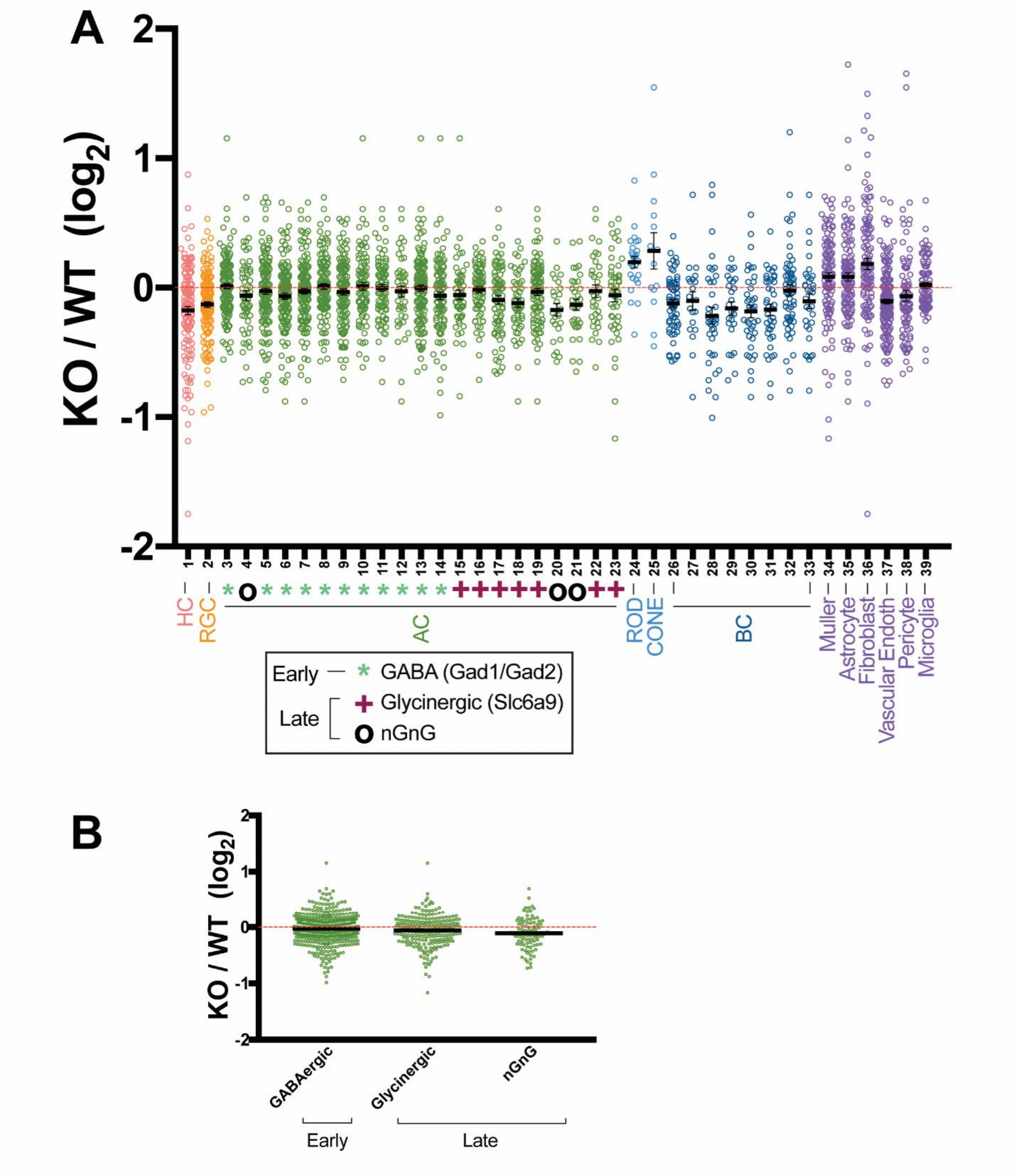


**Supplementary Figure S5:** Expression changes of cell type-specific gene sets. (**A**) Gene expression (*Mll1KO* vs *WT* [log2]) of cell-type-specific genes from 39 different retinal cell types, determined by drop seq (Macosko 2015) confirms overrepresentation of photoreceptor and glial gene expression. Subtype expression of Bipolar (BC) and Amacrine cell (AC) genes support a complex phenotype with some subtypes being more affected than others. (**B**) Overall, early-born GABA-ergic ACs are less affected than late-born non-GABA/non-Glycinergic (nGnG) ACs.

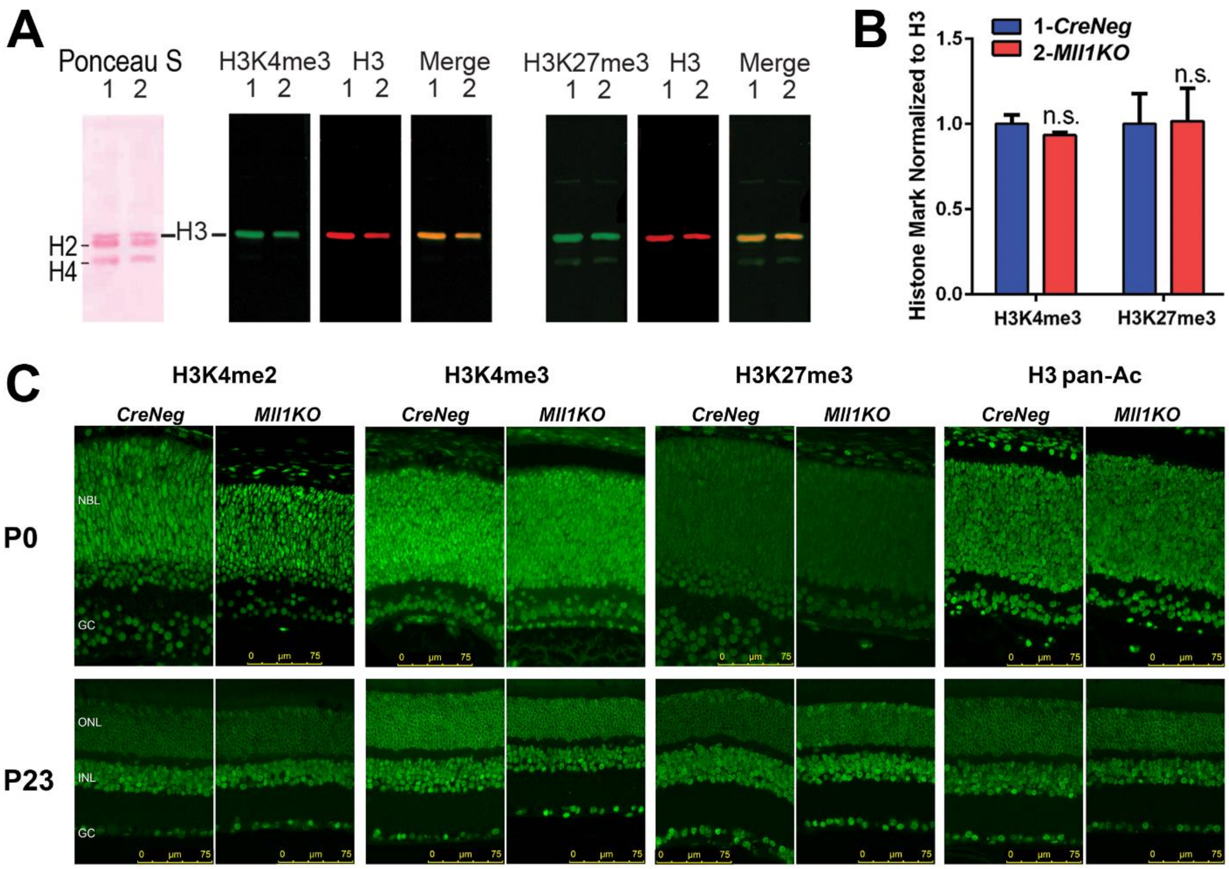


**Supplementary Figure S6:** Comparison of histone marks in *CreNeg* and *Mll1KO* retinas. (**A**) Histones were acid-extracted from P24 *CreNeg* (Lane 1) or *Mll1KO* (Lane 2) retinas as previously described (Hennig 2013). Western Blots were stained with Ponceau S for protein bands, then with the antibodies indicated (see Supplementary Table S2 for antibody information). “Merge” shows both channels together. (**B**) Quantification of band intensities, normalized to Histone H3. Results are presented as the mean+SEM of three experiments. (**C**) Histologic sections from P0 and P23 *CreNeg* and *Mll1KO* retinas were immunolabeled for the histone marks indicated. No obvious differences were seen between the different genotypes in the intensity, distribution, or nuclear pattern of staining at either age.

**Supplementary Table S1: *Mll1* PCR Primer Sequences**

| Designation | | Sequence | Location in NM_001081849 |
|---|---|---|---|
| **Primer Set 1** | qRT-PCR Forward | 5'- AGGAAGCCCAAGAAAGGACTC -3' | nt 10969-10989 |
| | qRT-PCR Reverse | 5'- AATCCCCAGCATCCGCAAAC-3' | nt 11148-11129 |
| **Primer Set 2** | Forward deletion | 5'-TGAGTACAACCCTAACGATGAGGAA -3' | nt 11349-11373 |
| | Reverse deletion | 5'- CGGAATCTCATGGGCATTG -3' | nt 11444-11426 |
| **Genotyping Primers** | Mll1-Flox_F | 5'- TCTCTGAAGTAAGCCTTTCTTAG -3' | (not in CDS) |
| | Mll1-Flox_R | 5'- CAGTGGACATTCCAACTCTTCAA -3' | (not in CDS) |

**Supplementary Table S2: Antibodies**

| Antibody | Source | Species | Marks |
|---|---|---|---|
| Activated Caspase-3 | R&D Systems AF835 | Rabbit | Apoptotic cells |
| AP-2-alpha (AP-2a) | DSHB 3B5 | Mouse | Amacrine cells |
| Brn3a | Millipore AB5945 | Rabbit | GC |
| Calbindin D-28K | Sigma C9848 | Mouse | HC, AC |
| Calbindin D-28K | Sigma C7354 | Rabbit | HC, AC |
| Calretinin | Millipore ab5054 | Rabbit | AC, HC |
| Chx10 | Exalpha X1180P | Sheep | Progenitors, pan-BP |
| CtBP2 | BD Biosciences 612044 | Mouse | Cones, ribbon synapses |
| Cyclin D1 | Sigma C7464 | Mouse | Proliferating cells |
| Cyclin D3 | Sigma AV03038 | Rabbit | Proliferating cells |
| Glutamine Synthetase | BD Biosciences 610517 | Mouse | Mueller glia |
| GlyT1 | Millipore AB1770 | Goat | Glycinergic AC |
| H3K4me2 | Abcam 7766 | Rabbit | Histone H3 di-methyl K4 |
| H3K4me3 | Millipore 07-473 | Mouse | Histone H3 tri-methyl K4 |
| H3K9me3 | Abcam 8898 | Rabbit | Histone H3 tri-methyl K9 |
| H3K27me3 | Upstate 07-449 | Rabbit | Histone H3 tri-methyl K27 |
| H3 pan-Ac | Millipore 106-599 | Rabbit | acetylated Histone H3 |
| Ki67 | BD Biosciences 550609 | Mouse | Proliferating cells |
| Lim1 (4F2) | DSHB AB 531784 | Mouse | HC |
| MLL1 | Bethyl A300-086A | Rabbit | MLL1 |
| Neurofilament (NF-M) | DSHB 2H3 | Mouse | Axons |
| Onecut 1 (HNF6) | Santa Cruz sc-13050 | Rabbit | HC, Cones |
| Onecut 2 | R&D AF6294 | Sheep | HC |
| p27Kip1 | BD 610241 | Mouse | Cycling cells, MG |
| Pax6 | DSHB Pax6 | Mouse | Progenitors, AC |
| Phospho-histone H3 | Milllipore 06-570 | Rabbit | Mitotic cells |
| PKCa | Sigma P5704 | Mouse | Rod BP |
| PNA-Rhodamine | Vector Labs RL-1072 | (lectin) | Cones |
| Prox1 | Millipore MAB5652 | Mouse | HC, AC, BP |
| Rhodopsin (RetP1) | Sigma O4886 | Mouse | Rods |
| Sall3 | Sigma HPA016656 | Rabbit | Cones, HC |
| VGluT1 | Millipore AB5905 | Guinea Pig | PR synaptic terminals |

**Supplementary Table S3. HC Gene PCR Primer Sequences**

| Gene Name (Refseq #) | Designation | Sequence | Location |
|---|---|---|---|
| ***Cx57* (NM_010289)**<br>(gap jct a10; Gja10) | Cx57 F1<br>Cx57 R1 | 5'- GGTTTGGACAGACAGATTAGG -3'<br>5'- GTGATGGGCTATTTTCTCCG -3' | nt 1251-1270 Exon 2<br>nt 1405-1386 Exon 2 |
| ***OC1* (NM_008262)**<br>(*Hnf6*) | Oc1 F1<br>Oc1 R1 | 5'- GGAAAGAGCAAGAACACGG -3'<br>5'- GATGAGGACGATGAACTGC -3' | nt 1491-1509; Exon 2<br>nt 1752-1694; Exon 2 |
| ***Prox1***<br>**(NM_008937)** | Prox1 F4<br>Prox1 R4 | 5'- CAGAAGGACTCTCTTTGTCAC -3'<br>5'- GCTGAACCACTTGATGAGC -3' | nt 2120-2140, Exon 2<br>nt 2355-2337, Exon 4 |

## REFERENCES

Hennig, A. H., Peng, G.-H., & Chen, S. Transcription coactivators p300 and CBP are necessary for photoreceptor-specific chromatin organization and gene expression. *PLoS ONE* **8**, e69721, doi:10.1371/journal.pone.0069721 (2013).

Macosko, E. Z. *et al.* Highly parallel genome-wide expression profiling of individual cells using nanoliter droplets. *Cell* **161**, 1202-1214, doi:10.1016/j.cell.2015.05.002 (2015).
